# A Comprehensive Network Integrating Signature Microbes and Crucial Soil Properties During Early Biological Soil Crust Formation on Tropical Reef Islands

**DOI:** 10.3389/fmicb.2022.831710

**Published:** 2022-03-17

**Authors:** Lin Wang, Jie Li, Si Zhang

**Affiliations:** ^1^CAS Key Laboratory of Tropical Marine Bio-Resources and Ecology, South China Sea Institute of Oceanology, Chinese Academy of Sciences, Guangzhou, China; ^2^Innovation Academy of South China Sea Ecology and Environmental Engineering, Chinese Academy of Sciences, Guangzhou, China

**Keywords:** biological soil crust, tropical reef island, biocrust formation, microbiome, geographical distribution pattern, enzyme activity

## Abstract

Biological soil crusts (BSCs/biocrusts), which are distributed across various climatic zones and well-studied in terrestrial drylands, harbor polyextremotolerant microbial topsoil communities and provide ecological service for local and global ecosystem. Here, we evaluated BSCs in the tropical reef islands of the South China Sea. Specifically, we collected 41 BSCs, subsurface, and bare soil samples from the Xisha and Nansha Archipelagos. High-throughput amplicon sequencing was performed to analyze the bacterial, fungal, and archaeal compositions of these samples. Physicochemical measurement and enzyme activity assays were conducted to characterize the soil properties. Advanced computational analysis revealed 47 biocrust-specific microbes and 10 biocrust-specific soil properties, as well as their correlations in BSC microbial community. We highlighted the previously underestimated impact of manganese on fungal community regulation and BSC formation. We provide comprehensive insight into BSC formation networks on tropical reef islands and established a foundation for BSC-directed environmental restoration.

## Introduction

Approximately 12% of Earth’s terrestrial surface is covered by biological soil crusts (BSCs/biocrusts), which provide ecosystem services and impact biogeochemical fluxes on a global scale (Grote et al., 2010; Castillo-Monroy et al., 2011; Elbert et al., 2012; Weber et al., 2015; Mogul et al., 2017; Maier et al., 2018). However, recent research estimated that biocrusts will decrease by approximately 25–40% within 65 years, mainly because of anthropogenic activities, highlighting the urgent need for rehabilitation and conservation (Rodriguez-Caballero et al., 2018).

Biological soil crusts harbor polyextremotolerant microbial topsoil communities including bacteria, archaea, fungi, lichen, and mosses, forming a model system for studying the assembly principles of microbial communities. Based on the dominating photoautotrophic organisms, biocrusts are categorized into the cyanobacterium-, lichen-, and moss-dominated types. Each type exhibits distinct colors and morphologies representing successional stages (Bowker et al., 2006b; Büdel et al., 2009; Weber et al., 2012). During the temporal development of BSCs, several key microbes have been found to stimulate pivotal events in the successional process. First, filamentous cyanobacteria (e.g., *Microcoleus vaginatus*) promote the initial surface soil colonization and moisture-holding capacity by producing polysaccharides (Mazor et al., 1996; Kuske et al., 2011; Pepe-Ranney et al., 2015). Subsequently, heterotrophic diazotrophs (e.g., Clostrideaceae and Proteobacteria) mediate N_2_-fixation in the subsurface of early successional BSCs, whereas heterocystous cyanobacteria (e.g., *Scytonema*, *Spirirestis*, and *Nostoc*) dominate N_2_-fixation in the mature crusts (Yeager et al., 2007, 2012; Pepe-Ranney et al., 2015). Together with environmental factors, these species increase the moisture and nitrogen content of the topsoil, generating a metabolic hub that increases microbial diversity and population density and further favors seed germination and vegetation establishment (Weber et al., 2016), so that contribute to the development and succession of the whole soil ecosystem (Hu and Liu, 2003; Lan et al., 2015; Couradeau et al., 2016). Understanding the construction process of microbial topsoil communities is valuable for modulating biocrust and ecosystem stability.

Geographically, BSCs are common to arid and semi-arid environments and are distributed among various climatic zones, ranging from the Arctic Circle to the Namib Desert in Africa (Büdel et al., 2009; Yoshitake et al., 2010; Williams et al., 2017). BSCs in arid and semiarid regions have been widely studied (Nagy et al., 2005; Gundlapally and Garcia-Pichel, 2006; Zhang B. et al., 2016; Mogul et al., 2017; Zhang et al., 2018), with Cyanobacteria, Proteobacteria, Actinobacteria, Bacteroidetes, and Acidobacteria identified as the major bacterial phyla. Moreover, high levels of endemism are present in global BSC microbial communities (Büdel et al., 2009; Lan et al., 2015). Such divergent biocrust microbial consortia are highly associated with local environmental factors, such as radiation intensity, topographic traits, soil structure, and soil types (Zaady et al., 2000; Lan et al., 2012, 2015). A previous study demonstrated that terrestrial dryland biocrusts improve soil quality by enriching unique patterns of nutrient and metal elements via their diverse microbial communities (Beraldi-Campesi et al., 2010). However, BSC microbial community formation and the interaction of the community with biotic and abiotic factors on tropical islands are not well-understood.

Located in the western Pacific Ocean, the South China Sea Islands encompass three archipelagos (i.e., Dongsha, Nansha, and Xisha) with coral reefs as the predominant structure (Li et al., 2013). Coral calcareous sand is the most important component of coral reefs, but it is not conducive to the natural establishment of vegetation. From the perspective of environmental and biological elements, the coral reefs are regarded as “deserts” in the ocean, which lacking real soil and freshwater resources, and have extreme environmental characteristics, such as high salt, alkali, temperature, and light. Meanwhile, because of their geographic isolation, climate cycling, and ecological amplitude, the islands in South China Sea are endowed with unique biodiversity (Sivaperuman et al., 2008). Notably, soil and underground freshwater resources support the ecosystems on coral reefs, the key to livability (Zhao and Wang, 2015; Werner et al., 2017; Han et al., 2020). Using biocrusts to change the “desert” state of tropical reef islands is reminiscent of their application in the desert ecosystem and extreme environment. However, BSCs on tropical reef islands have rarely been reported. Previous studies documented that in the tropical reef islands, biocrusts play an important role in N-cycling in coral sand soil, and contribute to soil stability to reduce soil loss (Collier et al., 2021; Wang et al., 2021). Thus, understanding the biocrust community endemism will provide mechanistic insight useful for environmental rehabilitation in the terrestrial areas of tropical reef islands.

Here, we aimed to answer the following questions: (1) What is the composition of the biocrust microbiome derived from coral calcareous sands on the tropical reef islands? (2) What are the core microbes and key environmental factors? (3) How do biocrust-enriched microbes associate with their microenvironment in biocrust formation? Specifically, we collected biocrust samples and their subsurface soil and bare soil counterparts at 4 islands across the Nansha (NS) and Xisha (XS) Archipelagos. High-throughput 16S rRNA gene (Bacteria and Archaea) and fungal internal transcribed spacer (ITS) region sequencing was performed to determine their microbial composition. Additionally, biotic and abiotic soil properties such as soil composition, chlorophyll content, and enzyme activities were measured. Next, integrative bioinformatic analysis was performed to identify key microbial operational taxonomic units (OTUs) enriched in biocrusts on the reef islands, highlight crucial environmental factors, and dissect the combinatorial contribution of microbes and their correlated soil properties to biocrust formation.

## Materials and Methods

### Sampling and Storage

The BSC samples were collected from the Nansha Archipelago and Xisha Archipelago, the South China Sea during May and June 2016. The islands are influenced by the tropical marine climate; the average temperatures of Nansha and Xisha Archipelagos are > 27°C and 26–27°C and their average annual precipitation levels are ∼2800 and ∼1500 mm, respectively. Notably, biocrusts in these areas were mainly the cyanobacteria-dominated type (Wang et al., 2021). At each sampling site, topsoil (BSCs, top 0–1 cm of biocrusts), sub-surface soil (BSC_sub, the following 1–3 cm of biocrusts), and bare soil (BS, top 0–1 cm of adjacent soil containing no visible signs of biocrusts or black-crusted BSCs) samples were collected using sterile spatulas. Due to the mobility of cyanobacteria, some light crusts are not visually detectable, thus possibly included in bare soil samples upon collection. All collected samples were at least 100 m apart, including BSC and bare soil samples. A total of 41 soil samples was collected, 8 BSC, 8 BSC_sub, and 8 BS samples in Nansha Archipelagos, 7 BSC, 7 BSC_sub, and 3 BS samples in Xisha Archipelagos.

Samples for soil properties assays were stored at 4°C. The samples used to evaluate the chlorophyll *a* (Chl *a*) were transferred in Sterile Sampling Opaque Bags (EPN-4590, TWIRL ‘EM, Quebec City, QC, Canada) and stored in the dark at −20°C. Samples for DNA extraction were stored in 15-mL conical centrifuge tubes and preserved in LifeGuard™ Soil Preservation Solution (MO BIO Laboratories, Carlsbad, CA, United States) according to the manufacturers’ instructions.

### Measurement of the Soil Physicochemical and Biological Properties

The physicochemical properties of the soil samples were measured according to the Industry Measurement Standard of China, including agricultural trade standards, forestry industry standards, and national environmental protection standards (Supplementary Table S1). Briefly, the pH values of samples were analyzed electrometrically (Acosta-Martínez et al., 2007). Soil organic carbon was measured by the dichromate oxidation method of Walkey and Black (Nelson and Sommers, 1982). Soil organic matter content was determined by the loss-on-ignition method after heating the fresh soil for 24 h at 600°C (Schulte and Hopkins, 1996). Soil available kalium using the 1 mol/L NH_4_oAc Leaching-flaming luminosity (Chen et al., 2007). Soil total nitrogen was determined using an Elementar Vario EL analyzer (Matejovic, 1995), and soil total phosphorus was determined by the H_2_SO_4_–HClO_4_ digestion method (Kuo, 1996). Soil available phosphorus was determined using the Olsen method with NaHCO3 as an extractant (Kuo, 1996). Soil available sulfur contents were measured as depicted by Williams and Steinbergs (1959). Total water-soluble salts were analyzed using methods described by the Nanjing Institute of Soil Research and CAS (1980). Soil available boron content was determined by the colorimetric method in a solution of an alcohol-acetic mixture with quinalizarin at 610 nm (Kashin, 2012). Soil NO_3_-N and NO_2_-N were determined colorimetrically as a combined value by the hydrazine sulfate reduction method (Rand et al., 1975), soil NH_4_-N content was measured using the phenohypochlorite method (Solórzano, 1969), soil NH_3_-N concentrations was measured colorimetrically on a segmented flow analyzer (AA3, Seal Analytical, Norderstedt, Germany) (Hu et al., 2019). Soil available Zn, Cu, Mn, K, Ca, and Fe was extracted with DTPA solution (Lindsay and Norvell, 1978), which consists of 0.005 M DTPA + 0.01 CaCl_2_.2H_2_O + 0.1 triethanolamine (TEA) with pH adjusted 7.3 0 ± 0.5, and determined by atomic absorption spectrophotometer from HITACHI, Japan.

Chlorophyll *a* content was measured as a proxy of photosynthetic biomass and to assess the developmental stage of biocrusts in this study (Yeager et al., 2004; Couradeau et al., 2016). Chl *a* was extracted from 2 g of soil samples by incubation in 10 mL acetone (80%, v/v) at 4°C for 20 h (Marker and Jinks, 1982; Wellburn, 1994). The Chl *a* content of the filtered solution was determined using a spectrophotometric method with absorbance measured at 665 and 750 nm. Subsequently, acid treatment using hydrochloric acid was incorporated into the final calculation to increase the accuracy of the results (Lorenzen, 1967; Mush, 1980).

The activities of key enzymes involved in phosphorus, carbon, nitrogen cycling, and peroxide degradation were measured. Specifically, the activities of soil β-glucosidase (S-β-GC), soil lipase (S-LPS), soil fluorescein diacetate hydrolase (S-FDA), soil alkaline protease (S-ALPT), soil urease (S-UE), soil alkaline phosphatase (S-AKP), and soil catalase (S-CAT) were determined using soil system assay kits (Solarbio LIFE SCIENCE, Beijing Solarbio Science & Technology Co., Ltd., Beijing, China) by the spectrophotometric method. Briefly, soil urease activity was determined using urea as the substrate and expressed as μg NH_4_^+^-N d^–1^ g^–1^ (Liu et al., 2014). Soil alkaline protease activity was measured by using casein as the substrate (Zhang et al., 2015). Soil catalase activity was determined by spectrophotometry via the measurement of hydrogen peroxide breakdown (Trasar-Cepeda et al., 1999). Soil alkaline phosphatase, soil lipase, and soil β-glucosidase activities were measured by colorimetric determination of the released *p*-nitrophenol (410, 400, and 410 nm, respectively), with *p*-nitrophenyl phosphate, *p*-nitrophenyl butyrate, and *p*-nitrophenyl β-D-galactoside as substrates (Zhang et al., 2015; Sofi et al., 2016). Soil fluorescein diacetate hydrolase activity was determined using fluorescein diacetate as substrate colorimetrically at 490 nm (An and Kim, 2009).

### DNA Extraction, Amplification and Sequencing

Total DNA was extracted using a HiPure Soil DNA Kit (Magen, Guangzhou, China) according to the manufacturer’s instructions. The concentration and purity of genomic DNA were determined using a NanoVuePlus Spectrophotometer (GE Healthcare, Little Chalfont, United Kingdom). The V4 region of the bacterial 16S rRNA gene (primer set: 515F 5′- GTGCCAGCMGCCGCGGTAA-3′, 806R 5′-GGACTACHV GGGTWTCTAAT-3′), ITS1 region of fungal ITS (primer set: ITS1f 5′-CTTGGTCATTTAGAGGAAGTAA-3′, ITS2 5′-GCT GCGTTCTTCATCGATGC-3′), and V4-V5 region of archaeal 16S rRNA gene (primer set: Arch519F 5′-CAGCCGCCGCGGTAA-3′, Arch915R 5′-GTGCTCCCCCGC CAATTCCT-3′) were amplified in triplicate from each sample DNA extract with dual indices and adapters. The products generated from standard thermocycling with an annealing temperature, including 53°C with 30 cycles for bacterial V4 and archaeal V4-V5 regions, and 53°C with 35 cycles for the ITS1 region, were pooled and sequenced at Magigene (Biological Technology Co., Ltd., Guangzhou, China) on an Illumina Hiseq 2500 platform (San Diego, CA, United States). The raw sequencing data have been deposited in the National Center for Biotechnology Information (Study accession number PRJNA560457).

After quality checking of the raw sequencing data using Trimmomatic (V0.33) (Bolger et al., 2014), the reads were merged using FLASH (Version 1.2.11) (Magoč and Salzberg, 2011). The flashed reads were processed using the Quantitative Insights into Microbial Ecology (QIIME) software package (Version 1.8.0) (Caporaso et al., 2011) and compared with Gold database (Haas et al., 2011) using the UCHIME algorithm (Edgar, 2016) to obtain effective tags (Bates et al., 2010a; Edgar et al., 2011; Bokulich et al., 2012). Next, Uparse software (Uparse v7.0.1001) (Edgar, 2013) was implemented to cluster the effective tags into OTU with a threshold of 97% sequence identity (Edgar, 2013). For each representative sequence, the silva (for 16S, Quast et al., 2012) and Unite (for ITS, Kõljalg et al., 2013) databases were used to annotate taxonomic information with a confidence threshold of ≥0.5.

### Statistical Analyses

Alpha diversity-related values, Chao1 (richness estimate) and Shannon (diversity index) estimates of microbial communities were calculated using QIIME (Version 1.8.0) (Caporaso et al., 2011). One-way analysis of variance and *post hoc* comparison using Tukey’s test was conducted to compare the soil properties of different types of soil samples and the alpha diversity-related values (SPSS version 18 software, SPSS, Inc., Chicago, IL, United States). Additionally, permutational multivariate analysis of variance (Anderson, 2001) was performed to determine the pairwise statistical significance of differences among the three groups (i.e., BSCs, BSC_sub, and BS, vegan package in R; Oksanen et al., 2015). We graphically depicted multivariate relationships of microbial communities based on the Bray–Curtis (Steinhaus) distance using non-metric multi-dimensional scaling (NMDS, vegan package in R; Kruskal, 1964; Oksanen et al., 2015). The function envfit from the R vegan package was used to fit environmental vectors onto the ordination (Oksanen, 2015).

Operational taxonomic units with differential abundance levels in BSCs vs. BSC_sub and BSCs vs. BS were identified based on a model using negative binomial distribution (DESeq2 package in R; Love et al., 2014). OTUs with fold-change of abundance within the top and bottom 5% as well as Benjamini–Hochberg-adjusted *P*-value < 0.01 were considered as significantly increased and decreased species, respectively (Love et al., 2014; Maier et al., 2018).

The niche breadth approach was applied to measure habitat specialization as described by Pandit et al. (2009; Logares et al., 2012) and using Levin’s niche breath index (Levins, 1968):


$$B\,j=\frac{1}{\sum_{i=1}^{N}P^{2}\,i\,j}$$

where *Bj* indicates the habitat niche breadth and *Pij* is the relative abundance of OTU*j* in a given habitat *i*. The average *B*-values were measured from the microbial community among all soil samples as an index of habitat niche breadth at the community level. OTUs with mean relative abundances < 2 × 10^–5^ were removed to avoid erroneous indication of specialists (Pandit et al., 2009). Additionally, OTUs with *B*-values > 10 and <1.5 were considered as habitat generalists and specialists, respectively, as they were within the outlier area of the B distribution (Supplementary Figure S1) (Logares et al., 2012; Luo et al., 2019). Complementing the niche breadth approach, INDicator VALues (INDVAL) analysis (labdsv package in R; Roberts, 2010) was used to determine the specialists for BSCs (Dufrene and Legendre, 1997). OTUs with significant (*P* < 0.05) INDVAL values of >0.3 among the specialists determined by niche breadth were considered as strict specialists for BSCs (Liao et al., 2016; Luo et al., 2019).

To identify the highly correlated OTU modules and their association with soil physicochemical and biological properties, we applied weighted gene co-expression network analysis (WGCNA) (wgcna package in R; Langfelder and Horvath, 2008). To adapt to the negative binomial distribution of the microbiomic datasets, we modified WGCNA by utilizing Bray Curtis dissimilarity to cluster the microbial communities. OTUs with highly similar relationships were classified as a module, revealing their interconnectivity. Eigengene networks were then applied to study the correlations of the OTU modules with the physicochemical and biological traits of soil samples (Langfelder and Horvath, 2007).

To visualize the correlations in the network interface, a correlation matrix was constructed by calculating all the possible pair-wise Spearman’s rank correlations between the key soil properties and the signature OTUs (Zheng et al., 2018). A correlation between two items was considered statistically robust if the absolute value of Spearman’s correlation coefficient (ρ) was > 0.35 and the *P* < 0.05, and the *P*-values were adjusted with a multiple testing correction using the Benjamini–Hochberg method (Benjamini et al., 2001; Li et al., 2015). We applied the vegan R package (Oksanen, 2015) to perform the network analysis, and used the Gephi interactive platform and Cytoscape (version 3.5.1) (Shannon et al., 2003; Li et al., 2015) to make the network visualization.

## Results

### Physicochemical and Biological Soil Properties

To extract soil properties indicative of the BSC formation process, we performed one-way analysis of variance followed by *post hoc* analysis to measure the statistical significance of 21 physicochemical and 8 biological (soil enzyme and Chl *a* content) soil properties. Compared to bare soil (BS) and BSC subsurface soil (BSC_sub), the pH values of biocrusts were significantly lower, whereas the nitrogen contents, including soil nitrate nitrogen (NO_3_-N) and soil ammonia nitrogen (NH_3_-N) as well as soil available manganese (Mn), were significantly higher in BSCs (Supplementary Figure S2 and Supplementary Table S2). All biological soil properties evaluated except for soil alkaline protease activity and soil lipase activity exhibited elevated levels or enhanced enzymatic activities in BSCs (Supplementary Figure S2 and Supplementary Table S4). In summary, the 10 soil properties (pH, Mn, NH_3_-N, NO_3_-N, Chl *a*, S-β-GC, S-CAT, S-FDA, S-AKP, and S-UE) are considered as key BSC soil features.

### Microbial Diversity and Composition

Next, we performed bacterial and archaeal partial 16S rRNA gene as well as fungal ITS1 sequencing to identify the microbial composition in the BSCs, BSC_sub, and BS soil samples. After quality filtering and the removal of potential chimeras, 2,732,516 bacterial, 2,854,485 fungal, and 1,437,029 archaeal merged sequences remained.

Alpha diversity values determined as the Chao1 and Shannon indices were applied to characterize the richness and diversity of the microbial communities in different soil types, respectively. The richness of bacterial species in BSCs was significantly higher than that in BS. Additionally, both the richness and diversity of fungal species were greater in BSCs and BSC_sub comparing to in BS. In contrast, the biocrusts richness, number of observed species, and diversity of archaeal species were significantly lower than those in BSC_sub (Supplementary Figure S3 and Supplementary Table S3).

**FIGURE 1:**
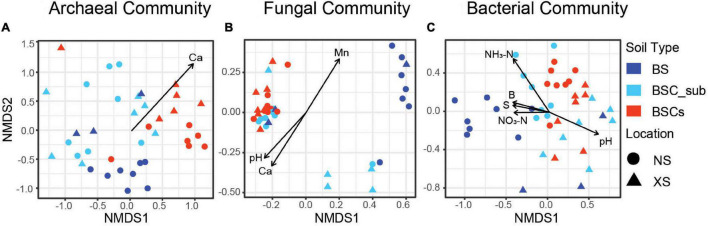
Dissimilarity of bacterial, fungal, and archaeal compositions in biocrusts comparing to sub-surface and bare soil samples. Ordination using NMDS is derived from Bray–Curtis dissimilarity and applied to analyze the archaeal **(A)**, fungal **(B)**, and bacterial **(C)** communities. Circle shape represents soils from Nansha Archipelagos whereas triangle represents Xisha Archipelagos. Different soil types are color coded. The function envfit from the R vegan package was used to fit environmental vectors onto the ordination (environmental factor significant correlation with NMDS, *P* < 0.05). NMDS, non-metric multidimensional scaling; NS, Nansha Archipelagos; XS, Xisha Archipelagos; BS, bare soil; BSC_sub, biocrust subsurface soil; BSCs, biocrusts; Mn, soil available manganese; NO_3_-N, soil nitrate nitrogen; NH_3_-N, soil ammonia nitrogen; B, soil available boron; S, soil available sulfur.

We observed significant differences in the microbiome in BSCs compared to BS and BSC_sub (Supplementary Table S5). Notably, NMDS analysis of bacteria, fungi, and archaea revealed a common pattern in which BSC samples were closely clustered, whereas BSC_sub and BS samples were relatively scattered (Figure 1). Only bacterial communities were distinct, while archaeal and fungal assemblages were not significantly different in BSCs collected from NS and XS (Supplementary Table S5). Except for the archaeal communities in BSC_sub, the microbiome showed a geographical-dependent distribution in BS and BSC_sub (Supplementary Table S5). Further, 7 soil parameters (i.e., pH, soil available boron, soil available sulfur, NO_3_-N, NH_3_-N, Ca, and Mn) were significantly correlated with the NMDS axes of the bacterial, archaeal, and fungal communities (Supplementary Table S6), which may contribute to soil microbial heterogeneity on these reef islands (Figure 1 and Supplementary Table S6).

### Biological Soil Crusts-Associated Operational Taxonomic Units

**FIGURE 2:**
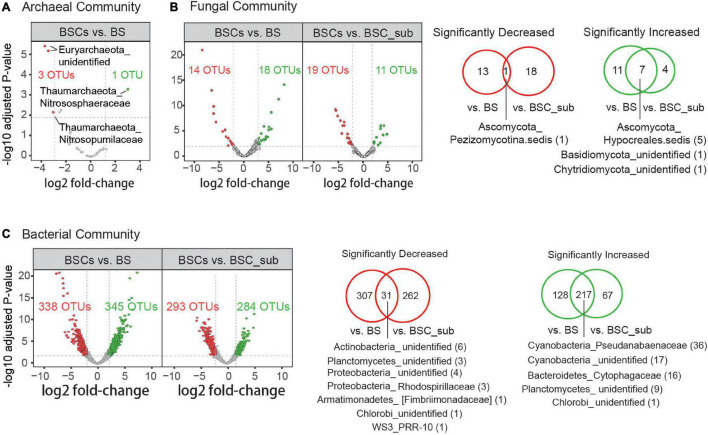
Archaeal, fungal, and bacterial OTUs with significantly altered abundance in BSCs comparing to BS and BSC_sub. Volcano plots represent the significantly increased (green) and decreased (red) OTUs (DESeq2, Benjamini-Hochberg adjusted *p*-value < 0.01, fold-change within top and bottom 5%) in archaeal **(A)**, fungal **(B)**, and bacterial **(C)** communities in BSCs comparing to BS and BSC_sub as indicated. Log2-transformed fold-change values of the relative abundance (*X*-axis) are plotted against adjusted *P*-values (unify *P*-value across the main text) (*Y*-axis). Venn diagrams illustrate the statistics of significantly increased and decreased OTUs corresponding to the right or bottom panel. Families with the top2 highest numbers of significantly co-altered OTUs are denoted as Phylum_Family (co-altered OTU number). BS, bare soil; BSC_sub, biocrust subsurface soil; BSCs, biocrusts.

To identify key OTUs showing significant alterations in abundance in BSCs compared to BSC_sub and BS, we performed differential analysis based on the negative binomial distribution (DESeq2) of the microbiomic datasets. As illustrated by the volcano plots, 217 and 31 bacterial OTUs as well as 7 and 1 fungal OTUs were commonly enriched and impoverished in BSCs, respectively (Figure 2). OTUs with significant alterations in BSCs were grouped by their families and phyla, and were listed by the fold-change in their abundance (Supplementary Figure S4). Notably, most Cyanobacteria (highly abundant bacterial phyla) and Chloroflexi (abundant bacterial phyla) were enriched in BSCs, whereas the relative abundances of Firmicutes (highly abundant bacterial phyla), Actinobacteria (abundant bacterial phyla), Nitrospirae (low abundance bacterial phyla), and WS3 (low abundance bacterial phyla) were decreased in BSCs (Supplementary Figure S4).

**FIGURE 3:**
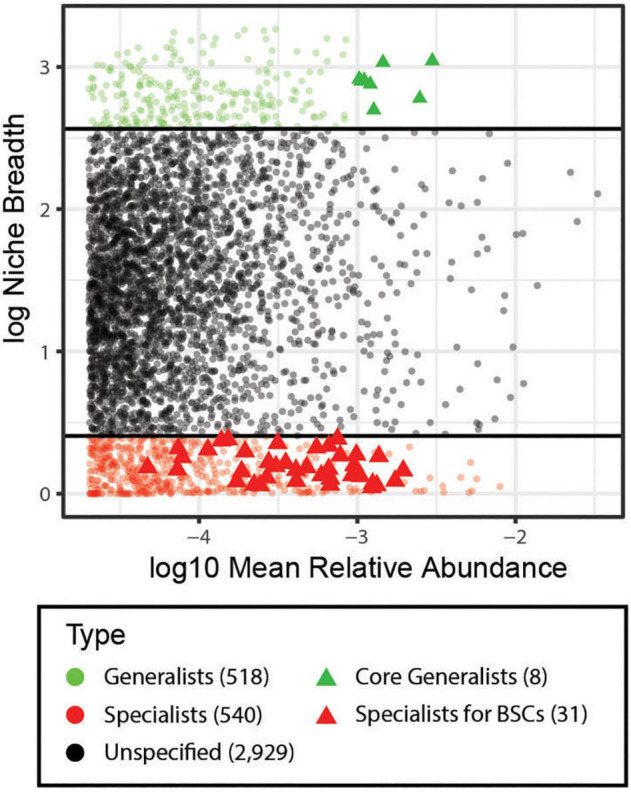
Analysis of habitat specialists and generalists. Niche breadth of OTUs identified across all sample types (BS, BSC_sub, and BSCs). Each symbol represents an OTU. OTUs that are present along a wider range of habitats have a higher niche breadth value and are considered habitat generalists (green), while OTUs with a niche breadth value < 1.5 are considered habitat specialists (red), and the black circles represent OTUs that could not be defined as generalists or specialists. Generalists with highest abundance (ubiquity cutoff: 94%, abundance cutoff: 4%) are considered as core generalists (green triangle). Specialists with significant INDVAL values (cutoff: >0.3) are specialists for BSCs (red triangle). Numbers outside of the square was represent the number of OTUs for each category. BS, bare soil; BSC_sub, biocrust subsurface soil; BSCs, biocrusts.

In analysis of the niche breadth, we identified 518 (13.0%) generalists and 540 (13.5%) specialists among all soil samples (Figure 3). Particularly, eight species were identified as core generalists (ubiquity cutoff: 94%, abundance cutoff: 4%, Figure 3), which were affiliated with Nitrososphaeraceae, Erythrobacteraceae, Ellin6067 (Betaproteobacteria), Ellin6075 (Chloracidobacteria), Ellin517 (Pedosphaerales), Chitinophagaceae, and Comamonadaceae. Through a combination of niche breadth and INDVAL analyses, 47 OTUs were identified as strict specialists associated with BSCs (Supplementary Table S7), 31 of which were strictly classified at the family level, including Cytophagaceae (2 OTUs), Flammeovirgaceae (2 OTUs), Cryomorphaceae (1 OTU), A4b (Anaerolineae, 2 OTUs), Chloroflexaceae (2 OTUs), Nostocaceae (1 OTU), Scytonemataceae (2 OTUs), Cyanobacteriaceae (1 OTU), Phormidiaceae (2 OTUs), Pseudanabaenaceae (6 OTUs), Lecanorales_fam_Incertae_sedis (1 OTU), Orbiliaceae (1 OTU), Helvellaceae (1 OTU), Pezizomycotina_fam_Incertae_sedis (2 OTUs), Glomerellaceae (1 OTU), Psathyrellaceae (1 OTU), Tulasnellaceae (2 OTUs), and Sebacinaceae (1 OTU) (Supplementary Figure S5 and Supplementary Table S7).

### Integrative Networks Analysis of Soil Properties and Microbiome

**FIGURE 4:**
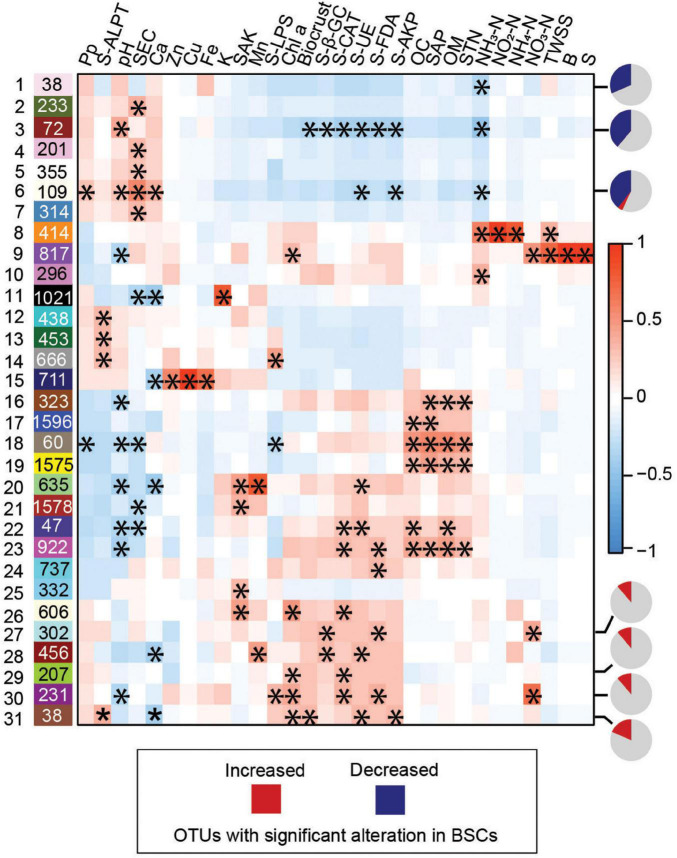
Weighted gene co-expression network analysis (WGCNA). The correlation among 53 modules (*Y*-axis) and 31 soil properties (*X*-axis) are illustrated in the heatmap. Positive correlation value is represented by red and negative by blue. The “*” in the cells are presented as microbial module significantly correlating with soil properties (*P* < 0.05). Pie charts on the right side of the indicated modules represent the percentage of significantly increased (red) and decreased (blue) OTUs in biocrust. Pp, Precipitation; B, Soil Available Boron; OM, Organic Matter; OC, Organic Carbon; SAP, Soil Available Phosphorus; SEC, Soil Exchangeable Calcium; SAK, Soil Available Kalium; K, Kalium; Ca, Calcium; Zn, Soil Available Zinc; Cu, Soil Available Copper; Fe, Soil Available Iron; Mn, Soil Available Manganese; TWSS, Total Water Soluble Salt; STP, Soil Total Phosphorus; S, Soil Available Sulfur; STN, Soil Total Nitrogen; NO_2_-N, Soil Nitrite Nitrogen; NO_3_-N, Soil Nitrate Nitrogen; NH_4_-N, Soil Ammonium Nitrogen; NH_3_-N, Soil Ammonia Nitrogen; Chl *a*, chlorophyll *a*; S-β-GC, soil β-glucosidase activity; S-LPS, soil lipase activity; S-FDA, soil FDA hydrolase activity; S-ALPT, soil alkaline protease activity; S-UE, soil urease activity; S-AKP, soil alkaline phosphatase activity; S-CAT, soil catalase activity.

**FIGURE 5:**
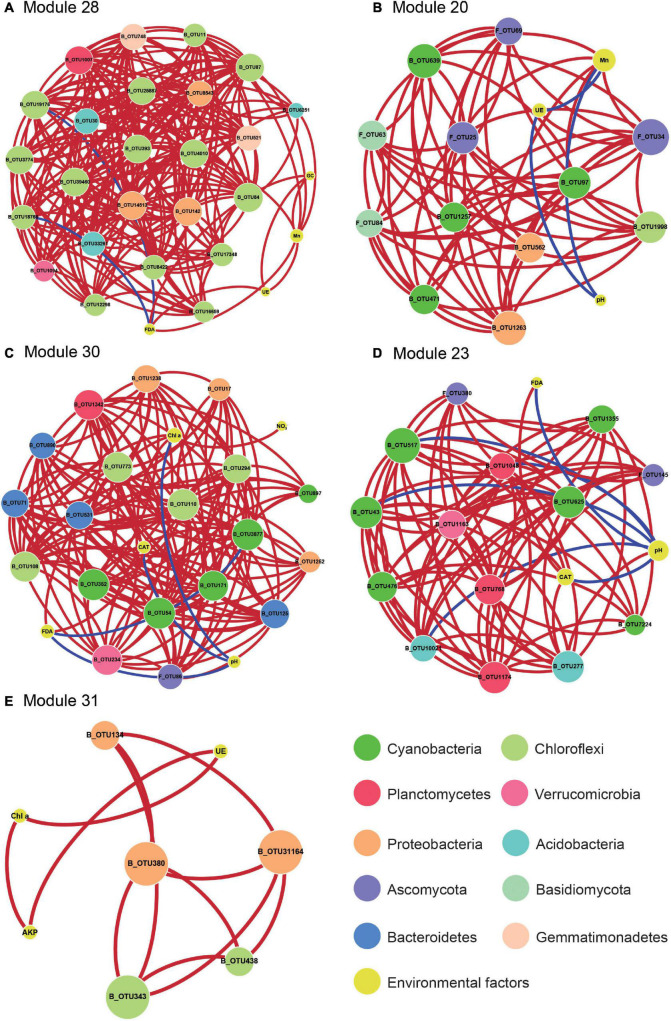
The network analysis revealing the correlation of the key properties with the signature OTUs. A correlation between two items was considered statistically robust if the absolute value of Spearman’s correlation coefficient (ρ) was > 0.35 and the *P* < 0.05. The nodes were the signature OTUs and the key properties, which from microbial modules, module 28 **(A)**, module 20 **(B)**, module 30 **(C)**, module 23 **(D)**, and module 31 **(E)**. OTUs were colored according to OTUs classification information. The size of each node was proportional to the number of connections. Graphics were generated in Cytoscape 3.5.1 using a circular layout. Chl *a*, chlorophyll *a*; GC, soil β-glucosidase activity; FDA, soil FDA hydrolase activity; UE, soil urease activity; CAT, soil catalase activity; NO_3_-N, soil nitrate nitrogen.

We evaluated whether variations in microbial compositions were associated with BSC-specific soil properties through WGCNA. The interconnectivity among all 26,917 OTUs was assessed through dissimilarity clustering, OTUs with highly similar occurrence, including frequency and relative abundance in the community, were classified as a module (Supplementary Figures S6A–C). Next, the correlations among the modules and soil properties were subsequently calculated by using eigenvalue (Langfelder and Horvath, 2007). Among the 31 modules and 30 soil properties showing at least one significant correlation (correlation coefficient *p*-value < 0.05; Figure 4), biocrust was significantly positively and negatively correlated with the module eigenvalues of module 31 and 3, respectively (Figure 4). Notably, the module 1, 3, 6, 27, 29, 30, and 31 strongly correlated with BSCs contained high percentages of significantly altered OTUs (among the top three negatively and top four positively correlated modules; Figure 4 and Supplementary Figure S7A). Additionally, the modules positively correlated with BSCs (except for module 31) comprised most BSC-specialists (Supplementary Figure S7B). Further, correlation analysis on biocrust-enriched OTUs and their microenvironmental properties within identified modules detailed the crucial associations (Figure 5). These observations uncovered the crucial microbial sub-communities in cyanobacterium-dominated BSCs on the tropical reef islands, South China Sea.

While WGCNA revealed the correlations among BSC-featured soil properties and microbial sub-communities, the relationship between key environmental factors and microbes remained unclear. We employed the co-occurrence network to integrate key soil properties representing BSC traits (i.e., pH, Mn, NO_3_-N, NH_3_-N, Chl *a*, S-β-GC, S-CAT, S-FDA, S-AKP, and S-UE) as well as signature OTUs comprising co-increased/decreased species (Figure 2), core generalists, and BSC-associated strict specialists. Notably, the key soil properties were significantly correlation with the microbial community and highly correlated with biocrusts (absolute value of correlation value > 0.5). Five BSC-associated microbial modules (i.e., module 20, 23, 28, 30, and 31), which were highly correlated with at least three key BSC soil features and at least one signature OTU, were selected to generate the co-occurrence network (Figure 5 and Supplementary Table S8).

The network in Figure 5 was used to visualize the correlation of key BSC features with the signature OTUs based on Spearman’s correlation coefficient (ρ) and *P*-value. In the Figure 5, the species that contain chlorophylls/bacteriochlorophylls or impact chlorophyll contents possess more complicated relationship in the network, which means that these OTUs play an important role in the network constructure. We observed that bacteria_OTU639 (Leptolyngbya), OTU11 (Phormidiaceae), OTU6251 (RB41), OTU87 (Cyanobacteria), OTU393 (Leptolyngbya), OTU1263 (Acetobacteraceae), OTU97 (Nostocales), and fungi_OTU25 (Pleosporales), OTU34 (Leprocaulon) were positively significant correlated with Mn that enhanced in biocrusts (Figures 5A,B). However, the key soil properties, i.e., pH and S-FDA, were negatively correlated with the signature OTUs that in the network (Figures 5A,C,D). Additionally, the biocrust-enhanced Chl *a* content was positively correlated with bacteria_108 (Anaerolineae), which was significantly enriched in BSCs (Figure 5C). Notably, in addition to bacteria members, fungi_OTU34 (Pleosporales) was observed to be associated with S-UE (Figure 5B).

## Discussion

We performed the first study on biocrusts by integrating the bacterial, fungal, and archaeal microbial communities. We observed that the microbial composition in biocrusts is distinct from that in bare soil. Specifically, the relative abundances of major microbial phyla in the BSCs, including Cyanobacteria, Chloroflexi, Ascomycota, and Thaumarchaetota, were significantly increased compared to in the BS (Figure 2). As the biocrusts collected from the South China Sea were mostly in the early successional stage, the enrichment of Cyanobacteria facilitates soil stabilization in the tropic reef islands, South China Sea (Mazor et al., 1996). Moreover, Cyanobacteria proliferation contributes to the cycling of organic matters and nutrients in biocrusts and promotes the recruitment of diverse microbial communities (Castenholz et al., 2001; DeFalco et al., 2001; Pendleton et al., 2003), facilitating the formation and development of BSC (Belnap and Lange, 2003; Garcia-Pichel and Wojciechowski, 2009). As one of the anoxygenic photosynthetic bacteria phyla, Chloroflexi lives phototrophically under anaerobic conditions (Hanada, 2019), and may be enriched in the below-crust portion of BSCs (Steven et al., 2013) to support carbon fixation. Ascomycota is a major fungal phylum in the cyanobacterium-dominated biocrusts. The enrichment of Ascomycota enhances fungal-loop formation, which translocate N from NH_4_^+^ over NO_3_^–^ to facilitate nitrogen cycling (Aanderud et al., 2018). Further, the major archaea phyla, Thaumarchaeota, is highly enriched in the BSCs and serves as an important biogeochemical agent of biocrust N cycling (Marusenko et al., 2013). Notably, Cyanobacteria and Thaumarchaeota were enriched in the BSCs compared to in BS and BSC_sub. Additionally, 17 Cyanobacteria, 4 Chloroflexi, and 10 Ascomycota OTUs were identified as strict specialists associated with BSCs, comprising of ∼66% of the strict specialist category (Figure 3), suggesting their substantial contributions to BSC formation and development.

Compared to BS, BSC exhibits higher homogeneity with BSC_sub in terms of microbial composition (Figure 1). Previous studies demonstrated that BSC microbial communities act as primary producers of local nutrients, transferring and cycling inorganic and organic soil components to the heterotroph communities in the subsurface (Dettweiler-Robinson et al., 2018; Maier et al., 2018). We propose that nutrient transfer and diffusion from the autotrophs in the BSCs to the heterotrophs in the BSC_sub led to the microbial and physicochemical similarity between BSCs and BSC_sub. It has also been reported that under dark dim light and moisture conditions, many subsurface populations of filamentous cyanobacteria migrate vertically to the surface; when sensing impending drought, these species return to their subsurface refuge (Garcia-Pichel and Pringault, 2001; Pringault and Garcia-Pichel, 2004). Thus, microbial vertical migrations, together with nutrient cycling between BSCs and BSC_sub, may contribute to the connectivity in microbial composition.

This is also the first comprehensive investigation of the spatial distribution of biocrusts in tropical reef islands. Although microorganisms (i.e., bacteria, fungi, and archaea) thrive together in the biocrusts, their geographical distribution patterns are distinct. Notably, bacterial communities exhibit significant differences in relative abundance between the Nansha Archipelagos and Xisha Archipelagos (Figure 1C and Supplementary Table S5). This observation is consistent with those of a previous study in the southwestern Idaho and Colorado Plateau, demonstrating that the geographic distribution impacts the BSC bacterial community (Steven et al., 2013; Blay et al., 2017). Additionally, this geographic heterogeneity in the microbial community was not observed for fungi or archaea, as neither exhibited differences in relative abundance between NS and XS (Figures 1A,B and Supplementary Table S5). In contrast, Steven et al. (2015) demonstrated a high level of spatial variability in the biocrust fungal community across the Colorado Plateau, except for a few conserved fungal lineages (predominantly belonging to order Pleosporales). As the samples from the Colorado Plateau were collected from sand soil and shale soil, whereas our NS and XS samples were uniformly collected from coral sand, the distinct physicochemical soil properties likely contribute to the different fungal distribution patterns in the BSCs observed by Steven et al. (2015). In agreement with our observation regarding the archaeal community spatial distribution, Soule et al. (2009) reported that archaeal populations were stable with no significant differences in diversity and maintain a high degree of conservation in the community composition in all types of biocrusts.

Further, we investigated the environmental drivers impacting the microbial communities in the tropic reef islands, South China Sea. Nitrogen contents, including nitrate and ammonia nitrogen, are highly enriched in the BSCs and significantly correlated with bacterial communities (Figure 1). As BSC-nourishing nutrients, nitrogen contents have been reported to determine the biocrust bacterial and fungal composition in coastal dunes (Schulz et al., 2016) and deserts (Zhang T. et al., 2016). Additionally, manganese is also enriched in the biocrusts and significantly associated with the fungal community. Consistently, a positive correlation between manganese availability and lichen/moss abundance in the biocrusts was previously established (Bowker et al., 2005). Moreover, fungi-mediated manganese accumulation and oxidation has also been reported (Thompson et al., 2005). These results highlight the underappreciated role of manganese in biocrust formation. Given the biological functions of manganese, we predict that manganese-oxidizing microbes in the BSCs utilize and accumulate manganese to facilitate photosynthesis (Barber, 2008), and nitrogen cycling (Zehr and Ward, 2002; Thompson et al., 2005). In addition, we observed that the pH is lower in the BSCs compared to in the BS and BSC_sub (Supplementary Figure S2) and significantly correlated with bacterial and fungal communities (Figure 1). In support of this observation, the pH has been shown to impact bacterial and fungal communities in biocrusts in the Gurbantunggut Desert (China), Intermountain West (United States), and Glacier Foreland (Norway) (Zhang B. et al., 2016; Blay et al., 2017; Borchhardt et al., 2019). Furthermore, our results demonstrated that the calcium contents were significantly correlated with the archaeal and fungal communities (Figure 1). Although it was not observed in this study, calcium contents are known to be highly correlated with the cyanobacterial and lichen communities in Arctic soil crusts (Dickson Land, Svalbard) and lichen-dominated biocrusts (Colorado Plateau, United States) (Bowker et al., 2006a; Pushkareva et al., 2015). In general, these results underscore the key physicochemical soil properties (i.e., NH_3_-N, NO_3_-N, pH, Mn, and Ca) contributing to BSC formation and development.

We investigated the impact of physicochemical soil features and microbial communities on BSC soil enzymatic activities. Our data demonstrated that the soil biological properties, including S-FDA, S-β-GC, S-AKP, S-UE, S-CAT, and Chl *a*, were significantly enhanced in the biocrusts (Supplementary Figure S2) and highly correlated with BSC microbial assemblages (Figure 4). Specifically, upon biocrust development, we observed that the pH value decreased from 9.6 to 8.8 (Supplementary Figure S2). Compared to the highly alkaline state in the BS, a relatively physiological soil pH in the BSCs may improve enzymatic performance, preserve enzyme conformation, and increase the solubility of substrates and cofactors for most enzymes (Quiquampoix, 2000). In addition, manganese was enriched in the biocrusts. Manganese is known to aid in chlorophyll synthesis and photosynthesis (Schmidt et al., 2016) and facilitate enzymatic activities of β-glucosidase (Olajuyigbe et al., 2016), alkaline phosphatase (Fitt and Peterkin, 1976), and manganese catalase (Whittaker, 2012). The combinatorial role of manganese-enhanced photosynthesis and enzymatic activation may contribute to BSC development. Elevation of nitrogen contents was also observed in our study and likely stimulates microbial community development and the energy supply for enzyme production (Fontaine et al., 2003; Yuan and Yue, 2012).

In addition to the physicochemical soil properties, the microbial abundance and composition have been reported to impact BSC enzymatic activities (Bates et al., 2010b; Castillo-Monroy et al., 2011). In this study, WGCNA analysis showed that several OTU modules were strongly correlated with soil enzyme activities. Further, network-based analysis revealed that seven microbial species significantly correlated with the critical soil enzyme activities (Figure 5); among them, five belong to the phylum Cyanobacteria. This is consistent with the prediction that Cyanobacteria significantly alter soil enzymatic activities (de Caire et al., 2000; Zhang et al., 2012).

According to a previous study, the earliest stage of biocrust formation in drylands is soil surface stabilization via filamentous Cyanobacteria (Scott, 1982; Büdel et al., 2016). In this study, 39.2% of nodes (key OTUs) were belong to the phylum Cyanobacteria according to network analysis (Figure 5), to affect BSC formation possibly by producing exopolysaccharides (Bellezza et al., 2003; Felisberto and Souza, 2014), stabilizing erodible substrates (Garcia-Pichel and Wojciechowski, 2009), and fixing CO_2_. Moreover, the BSC-specific bacteria_OTU17 (Erythrobacteraceae), OTU142 (Rubellimicrobium), OTU380 (Caulobacteraceae), OTU562 (Acetobacteraceae), OTU1263 (Acetobacteraceae), OTU14513 (Sphingomonadaceae), OTU31164 (Sphingomonadaceae), OTU8543 (Sphingomonadaceae), and OTU134 (*Sphingomonas*), account for 12.2% of the key OTUs, were potentially group of aerobic anoxygenic phototrophic bacteria (Figure 5), which could promote the development of BSC in drylands (Tang et al., 2018, 2021). Additionally, the bacterial family Sphingomonadaceae has been reported to produce exopolysaccharides and synthesize bacteriochlorophyll, assisting in soil particle bonding and increasing the biomass of the biocrusts (White et al., 1996; Tonon et al., 2014). Taken together, our analysis suggests that these two groups are critical for BSC formation and development in the tropic reef islands, South China Sea, possibly by enhancing BSC soil stabilization and biomass. In the network analysis, we also observed that many species, most of which contains chlorophylls/bacteriochlorophylls, were significantly correlated with the soil pH or Mn (Figure 5). This is mainly due to the fact that Mn and soil pH could regulate chlorophyll synthesis and photosynthesis rate (Quiquampoix, 2000; Schmidt et al., 2016), which in turn leads to the enrichment of related species in the biocrusts. These results demonstrate that BSC-enriched microorganisms, their biochemical properties, together with BSC-associated environmental factors, generate a multi-level network relationship that modulates the biocrust formation and development in the tropic reef islands, South China Sea.

## Conclusion

We first comprehensively investigated the microbiome composition in biocrusts on tropical reef islands and observed cyanobacterium-dominant characteristics in the early stage. Moreover, the microbiome in BSCs was distinct from that in bare soil and beneath the soil. The geographical distribution pattern of the bacterial community differed from the fungal and archaeal communities in BSCs on the tropical reef islands. In addition to geographical isolation, the biocrust microbial community was affected by soil properties including pH, soil available sulfur, soil available boron, soil available manganese, calcium content, soil nitrate nitrogen, and soil ammonia nitrogen. We identified 518 generalists and 540 specialists among all soil samples. A total of 47 OTUs was identified as strict specialists associated with BSCs. Further, we revealed the correlations of the signature species and soil properties in BSC microbial community. This study improves the understanding of the initiation and process of biocrust development on tropical reef islands, and the effects of microbes on this process.

## Data Availability Statement

The datasets presented in this study can be found in online repositories. The names of the repository/repositories and accession number(s) can be found in the article/Supplementary Material.

## Author Contributions

LW performed the field investigation, laboratory research, further data analyses, and prepared the manuscript. JL and SZ contributed to the discussion and analysis of data and finalization of the manuscript, and supervised the research. All authors carried out the study design, read and approved the final manuscript.

## Conflict of Interest

The authors declare that the research was conducted in the absence of any commercial or financial relationships that could be construed as a potential conflict of interest.

## Publisher’s Note

All claims expressed in this article are solely those of the authors and do not necessarily represent those of their affiliated organizations, or those of the publisher, the editors and the reviewers. Any product that may be evaluated in this article, or claim that may be made by its manufacturer, is not guaranteed or endorsed by the publisher.
