# Supplementary material for: A Comprehensive Network Integrating Signature Microbes and Crucial Soil Properties During Early Biological Soil Crust Formation on Tropical Reef Islands
Source: Front Microbiol. 2022 Mar 17;13:831710. doi: 10.3389/fmicb.2022.831710 (PMC8969229; doi:10.3389/fmicb.2022.831710)
Supplement: Supplementary file 1 [file Data_Sheet_1.docx]

**A Comprehensive Network Integrating Signature Microbes and Crucial Soil Properties during Early Biological Soil Crust Formation on Tropical Reef Islands**

Lin Wang^1^, Jie Li^1, 2*^, Si Zhang^1, 2*^

*Corresponding author.

E-mail address: lijietaren@scsio.ac.cn (Jie Li); Tel: +86 20 89023105

zhsimd@scsio.ac.cn (Si Zhang); Tel: +86 20 89023103

^1^ CAS Key Laboratory of Tropical Marine Bio-resources and Ecology, South China Sea Institute of Oceanology, Chinese Academy of Sciences, Guangzhou 510301, PR China

^2^ Innovation Academy of South China Sea Ecology and Environmental Engineering, Chinese Academy of Sciences, Guangdong 510301, China

**This file includes:**

Figs. S1 to S7

Tables S1 to S8


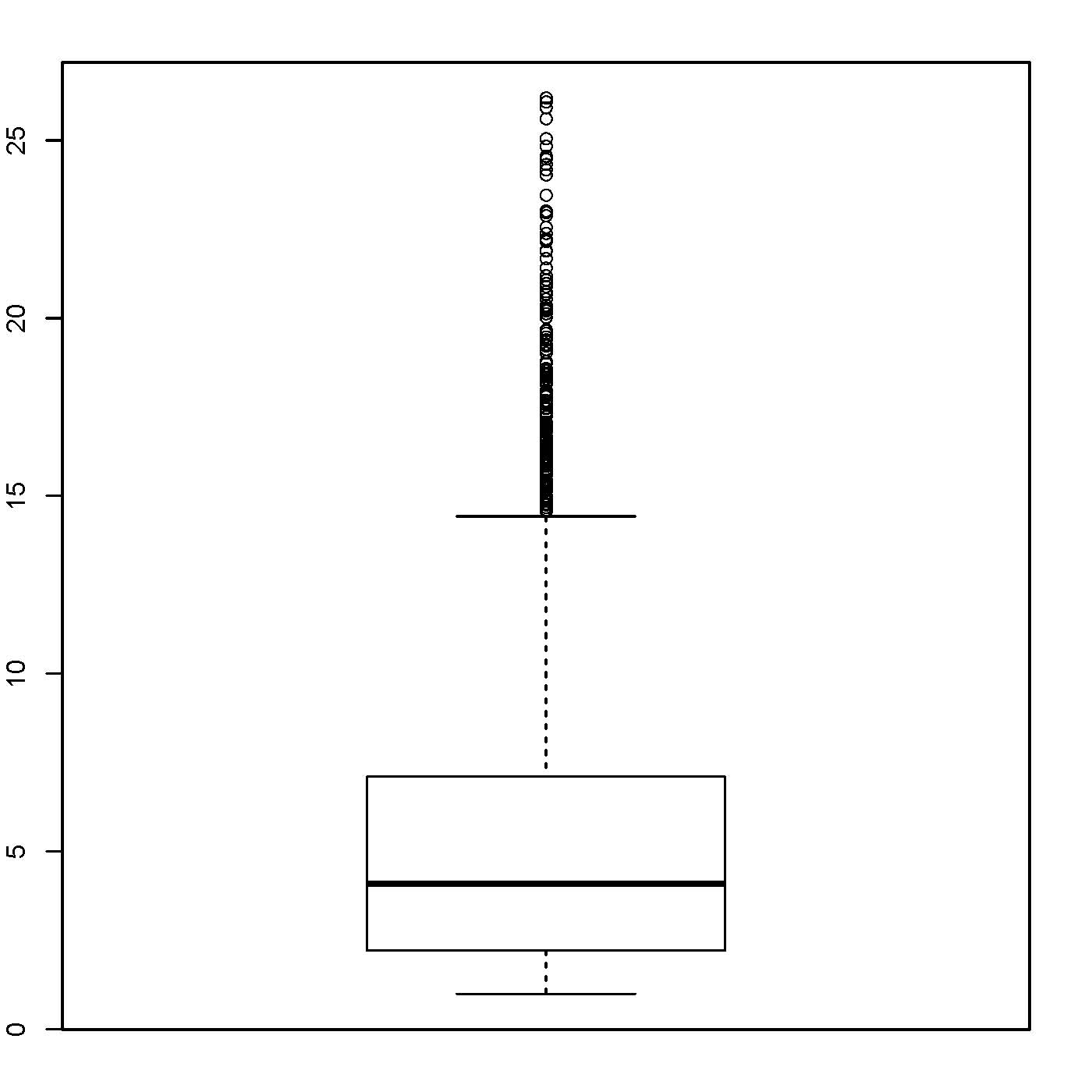


Fig. S1 B-value boxplot determining the B-value cut-offs. OTUs with a B-value >13 and <1.5 are considered as habitat generalists and specialists, respectively, as they are within the outlier area of the B distribution.


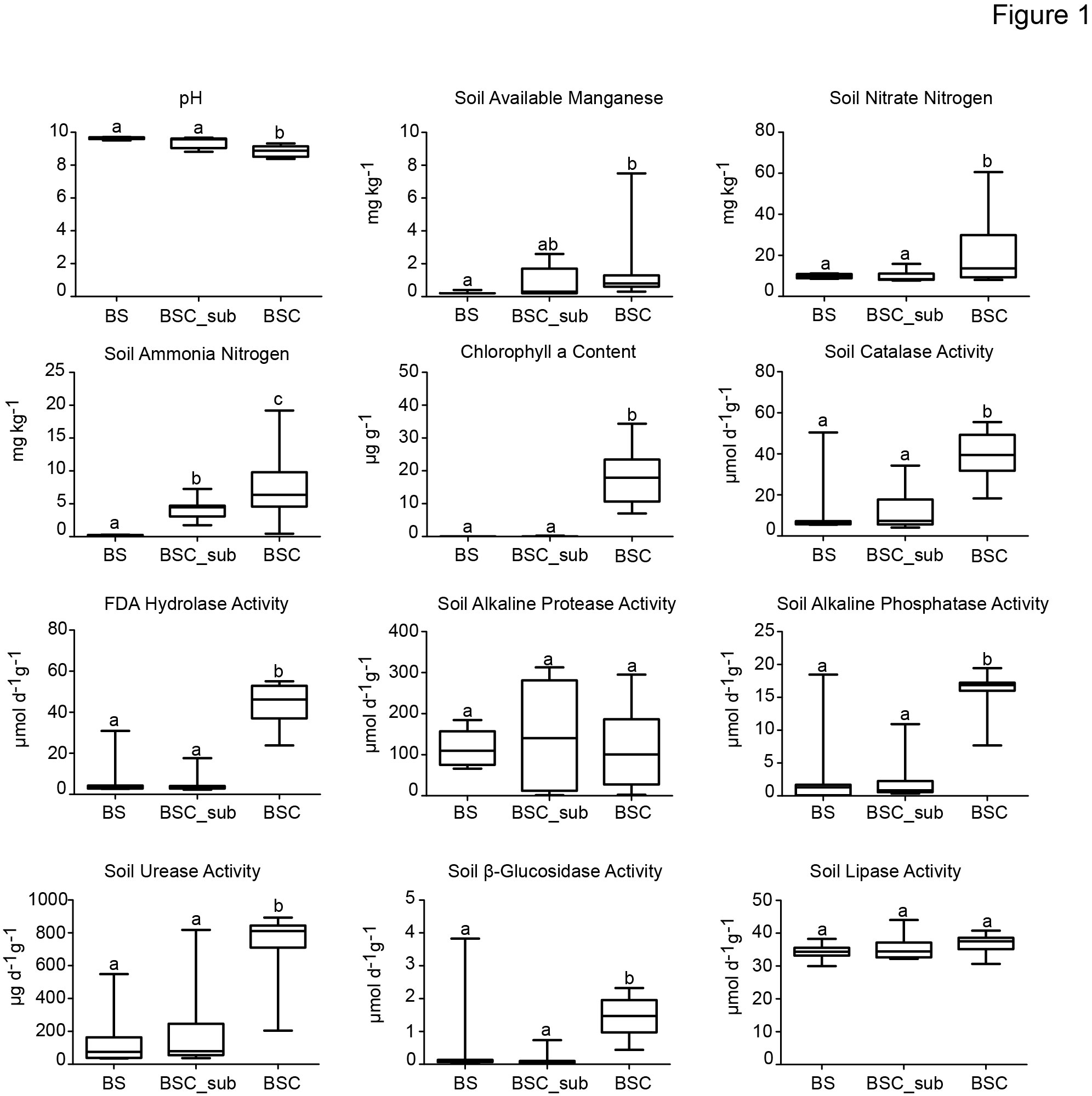


Fig. S2 Alteration of physicochemical and biological soil properties in the biocrusts samples. Four physicochemical soil properties (i.e., pH, soil available manganese, soil nitrate nitrogen, and soil ammonia nitrogen) and eight biological soil properties (i.e., chlorophyll a contents, soil lipase, soil alkaline protease, soil urease, soil catalase, soil alkaline phosphatase, soil β-glucosidase and soil FDA hydrolase) in different types of soil samples (BS, BSC_sub and BSC) are presented in the boxplot. Boxes limit the 25th- and 75th percentile with the median presented as line inside. Error bars present the 1st and 99th percentile and outliers are shown as dots below and above. Significant differences (P < 0.05) are marked by different letters. Abbreviations: bare soil (BS), BSC subsurface soil (BSC_sub), and biocrust (BSC).


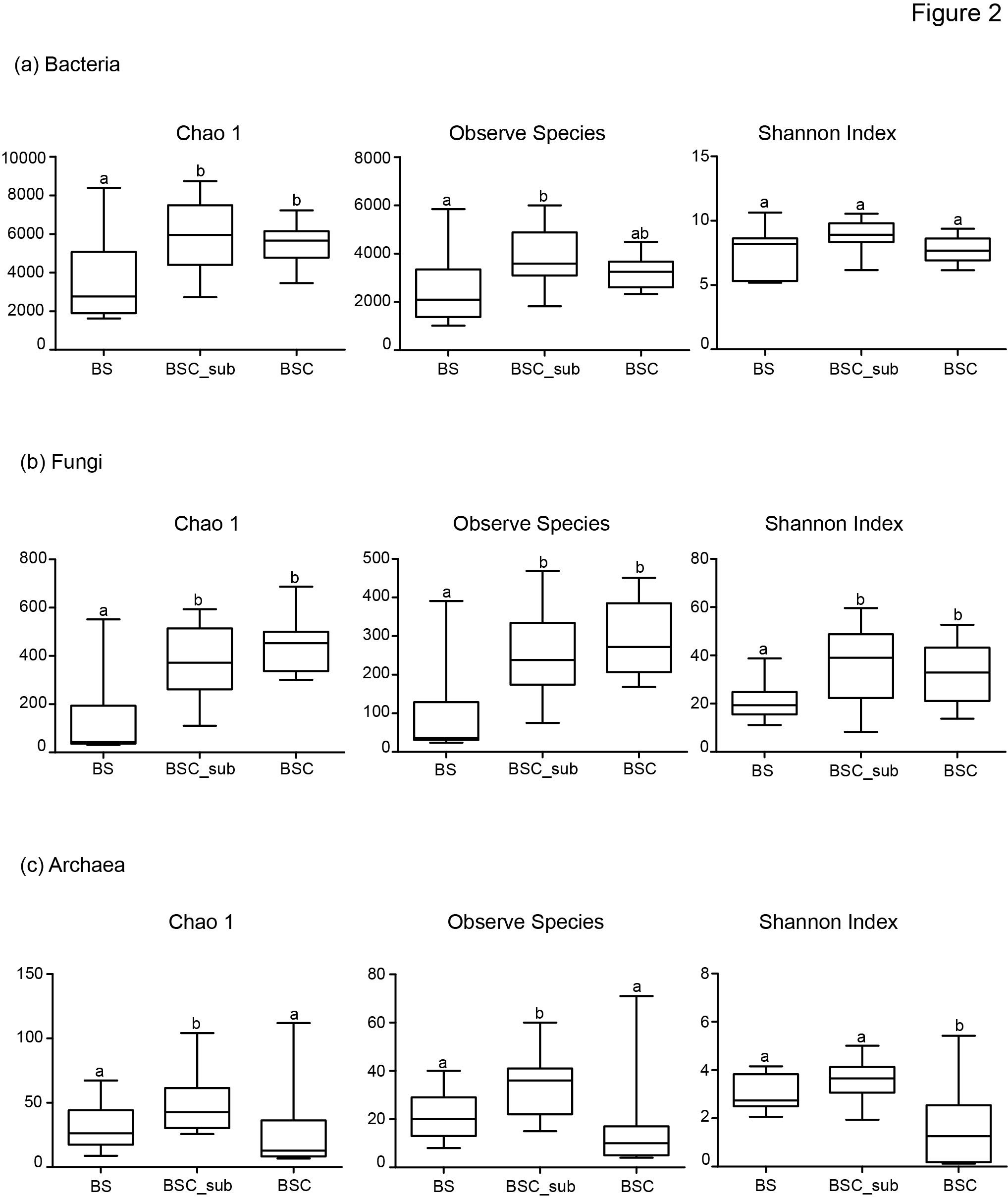


Fig. S3 Alteration of richness and diversity of bacterial, fungi and archaea communities in the biocrust samples. The boxplot demonstrates the alpha diversity parameters, Chao1, observed-species, and Shannon index, across three types of microbial communities, bacteria (A), fungi (B), and archaea (C). Boxes limit the 25th- and 75th percentile with the median presented as line inside. Error bars present the 1st and 99th percentile and outliers are shown as dots below and above. Significant differences (P < 0.05) are marked by different letters. Abbreviations: bare soil (BS), biocrust subsurface soil (BSC_sub), and biocrust (BSC).


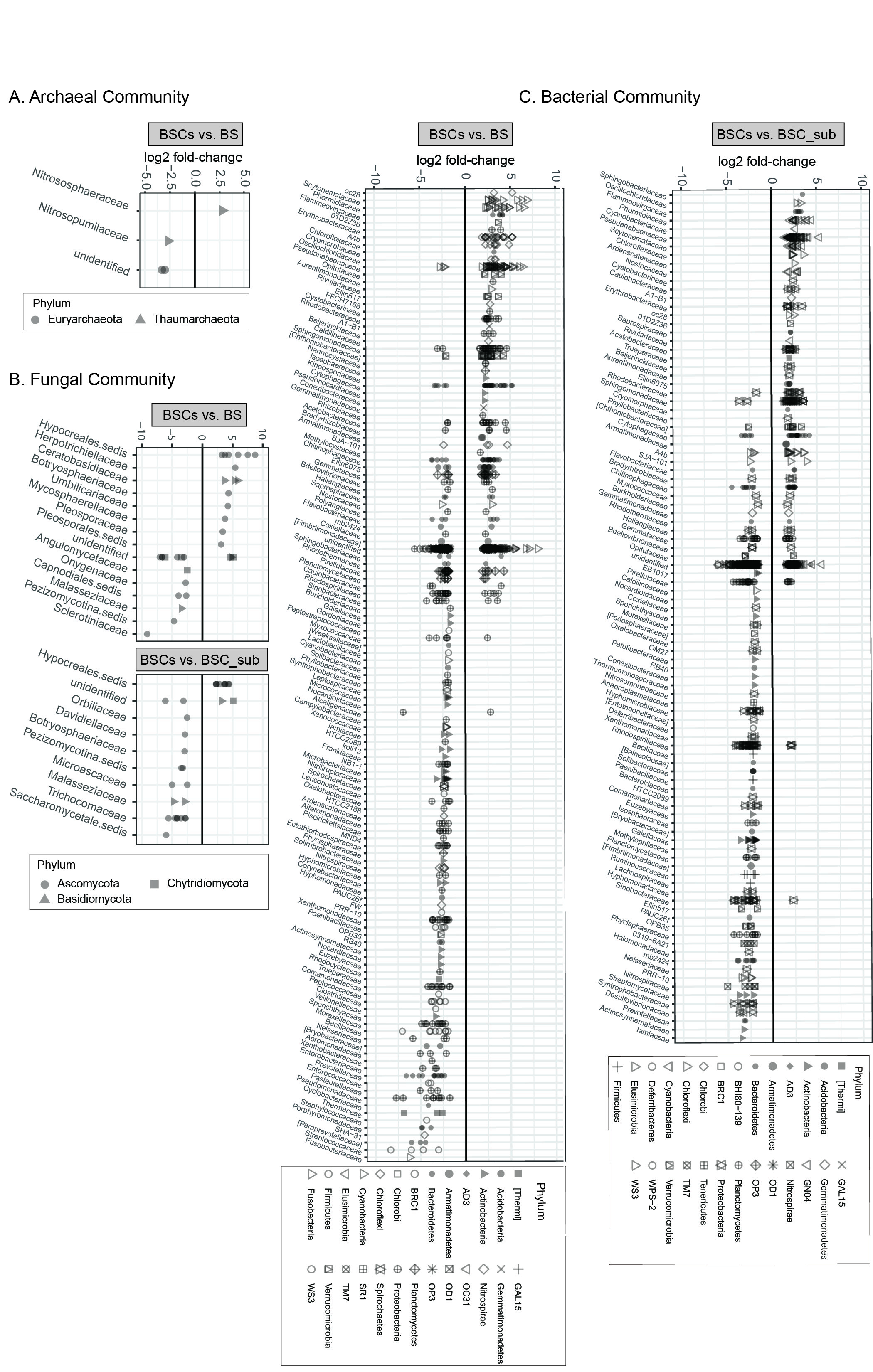


Fig. S4 Significantly altered (DESeq2, Benjamini–Hochberg adjusted P < 0.01, fold-change within top and bottom 5%) archaeal (A), fungal (B), bacterial (C) OTUs at family level in BSCs compared to indicated soil types (bare soil, sub-surface soil). Each symbol represents an OTU, whereas the shape of a symbol is determined by the phylum. The family of a OTU is plotted on the X-axis, and the log2-transformed fold-change value shown on the Y-axis. Abbreviations: bare soil (BS), biocrusts subsurface soil (BSC_sub), and biocrusts (BSCs).


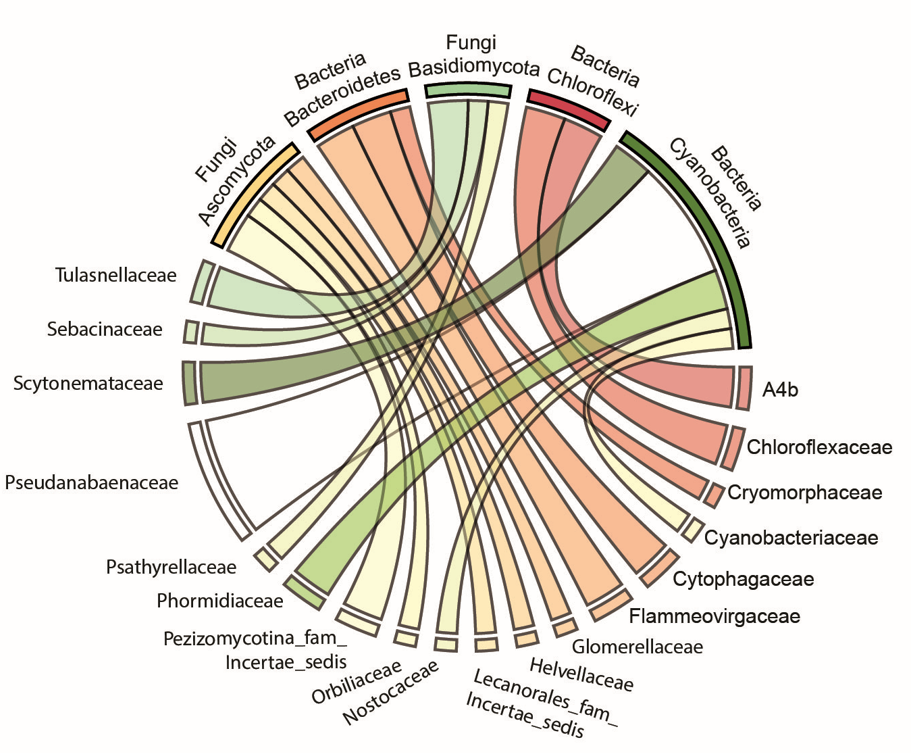


Fig. S5 Analysis of habitat specialists and generalists. Circos plot show taxonomic assignment for OTUs identified as specialists for biocrusts.


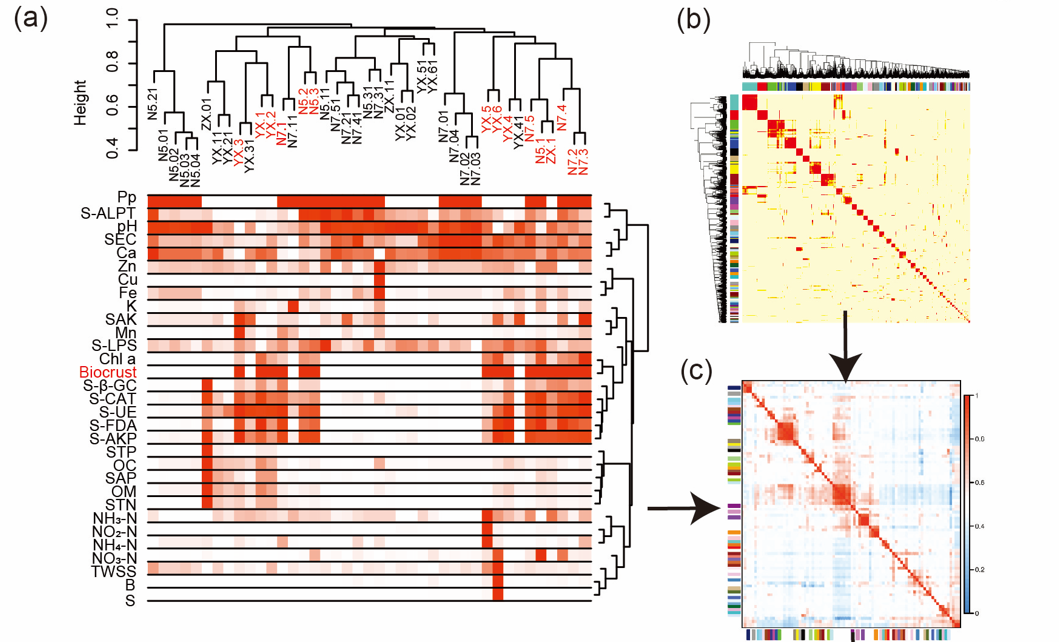


Fig. S6 Weighted Gene Co-expression Network Analysis (WGCNA). (a) OTU-based sample dissimilarity dendrogram with heatmap is demonstrated with soil properties clustered and sorted according to their dissimilarity. (b) The interaction pattern of the relative abundanceamong 26917 OTUs are presented as 53 modules in the heatmap based on topological overlap matrix. Degree of overlap is represented by the color shade: darker color represents higher overlap and lighter color represents lower overlap. (c) The disimilarity clustering among 53 modules and 31 soil properties are illustrated in the heatmap. Abbreviations: Precipitation (Pp), Soil Available Boron (B), Organic Matter (OM), Organic Carbon (OC), Soil Available Phosphorus (SAP), Soil Exchangeable Calcium (SEC), Soil Available Kalium (SAK), Kalium (K), Calcium (Ca), Soil Available Zinc (Zn), Soil Available Copper (Cu), Soil Available Iron (Fe), Soil Available Manganese (Mn), Total Water Soluble Salt (TWSS), Soil Total Phosphorus (STP), Soil Available Sulphur (S), Soil Total Nitrogen (STN), Soil Nitrite Nitrogen (NO_2_-N), Soil Nitrate Nitrogen (NO_3_-N), Soil Ammonium Nitrogen (NH_4_-N), Soil Ammonia Nitrogen (NH_3_-N), chlorophyll a (Chl a), soil β-glucosidase activity (S-β-GC), soil lipase activity (S-LPS), soil FDA hydrolase activity (S-FDA), soil alkaline protease activity (S-ALPT), soil urease activity (S-UE), soil alkaline phosphatase activity (S-AKP), soil catalase activity (S-CAT).


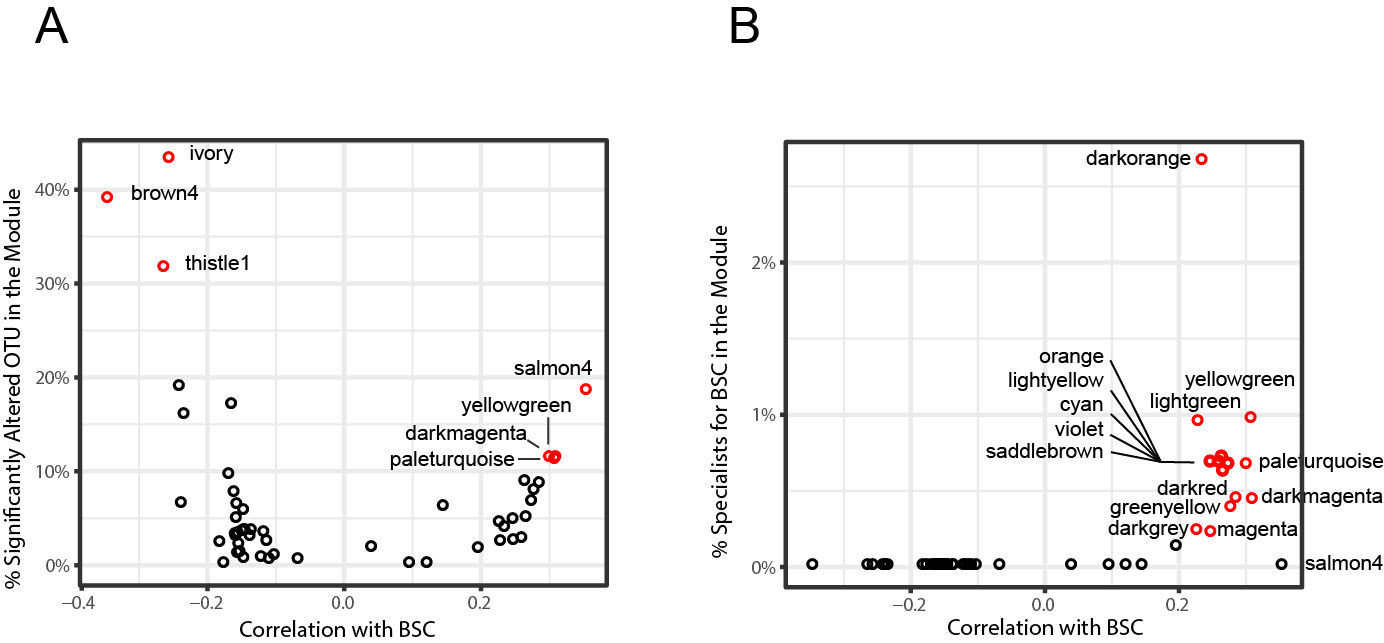


Fig. S7 Correlation between the modules with high percentage of featured OTUs and modules highly associated with BSC. X-axis of the dotplot represents the percentage of significantly altered OTUs (A) and the percentage of BSC associated specialists for indicated modules, whereas Y-axis represents the correlation with BSC. Red circle denotes the modules with high percentag of featured OTUs. Modules with highest percentages of significantly altered OTUs are mostly those with highest correlation with BSC. Meanwhile, modules with highest percentages of BSC specialists are mostly those with highest positive correlation with BSC. Abbreviations: Biological soil crust (BSC).

**Table S1 Test method for soil properties and measuring instrument**

| **Soil Property** | **Test Method** |
| --- | --- |
| Soil Available Boron | NY/T 149-1990, Agricultural trade standards of PRC |
| Organic Matter | NY/T 1121.6-2006, Agricultural trade standards of PRC |
| Organic Carbon | HJ 615-2011, National environmental protection standards of PRC |
| Soil Available Phosphorus | HJ 704-2014, National environmental protection standards of PRC |
| Soil Exchangeable Calcium | NY/T 1121.13-2006, Agricultural trade standards of PRC |
| Soil AvailableKalium | NY/T 889-2004, Agricultural trade standards of PRC |
| Kalium | NY/T 87-1988, Agricultural trade standards of PRC |
| Calcium | NY/T 296-1995, Agricultural trade standards of PRC |
| Soil Available Zinc | NY/T 890-2004, Agricultural trade standards of PRC |
| Soil Available Copper | NY/T 890-2004, Agricultural trade standards of PRC |
| Soil Available Iron | NY/T 890-2004, Agricultural trade standards of PRC |
| Soil Available Manganese | NY/T 890-2004, Agricultural trade standards of PRC |
| Total Water-Soluble Salt | NY/T 1121.16-2006, Agricultural trade standards of PRC |
| Soil Total Phosphorus | HJ 632-2011, National environmental protection standards of PRC |
| Soil Available Sulphur | NY/T 1121.14-2006, Agricultural trade standards of PRC |
| Soil Total Nitrogen | NY/T 53-1987, Agricultural trade standards of PRC |
| Soil Nitrite Nitrogen | HJ 634-2012, National environmental protection standards of PRC |
| Soil Nitrate Nitrogen | HJ 634-2012, National environmental protection standards of PRC |
| Soil Ammonium Nitrogen | LY/T 1228-2015, Forestry industry standards of PRC |
| Soil Ammonia Nitrogen | HJ 634-2012, National environmental protection standards of PRC |

**Table S2 Soil characteristics from Nansha islans and Xisha islands, South China Sea**

|  | **N5.1** | **N5.2** | **N5.3** | **N7.1** | **N7.2** | **N7.3** | **N7.4** | **N7.5** | **YX.1** | **YX.2** |
| --- | --- | --- | --- | --- | --- | --- | --- | --- | --- | --- |
| **pH** | 8.51 | 9.12 | 8.88 | 8.73 | 9.15 | 8.91 | 9.04 | 9.22 | 8.42 | 8.59 |
| **Soil Available Boron** | 0.042 | 0.019 | 0.015 | 0.028 | 0.029 | 0.093 | 0.087 | 0.018 | 0.01 | 0.018 |
| **Organic Matter** | 15 | 9.6 | 13.2 | 11.1 | 8.38 | 9.9 | 8.4 | 7.98 | 45.2 | 35 |
| **Organic Carbon** | 0.82 | 0.48 | 0.68 | 0.54 | 0.32 | 0.56 | 0.54 | 0.54 | 2.59 | 1.84 |
| **Soil Available Phosphorus** | 76.9 | 9.2 | 9.3 | 21.4 | 16.7 | 16.3 | 15.8 | 11.4 | 147 | 151 |
| **Soil Exchangeable Calcium** | 59.2 | 67.2 | 38.1 | 27.7 | 51 | 72.8 | 82.6 | 91.1 | 25.5 | 55.5 |
| **Soil Available Kalium** | 18.2 | 14.6 | 8.7 | 28 | 12.2 | 112 | 65.2 | 6.2 | 10.7 | 12.2 |
| **Kalium** | 3300 | 762 | 1700 | 1100 | 364 | 2300 | 1500 | 188 | 1100 | 349 |
| **Calcium** | 168 | 95.7 | 85.7 | 38.8 | 269 | 255 | 221 | 277 | 308 | 263 |
| **Soil Available Zinc** | 10.7 | 7.26 | 2.11 | 2.56 | 0.36 | 5.92 | 0.8 | 0.36 | 0.66 | 7.69 |
| **Soil Available Copper** | 0.7 | 0.17 | 0.41 | 0.17 | 0.25 | 0.11 | 0.51 | 0.25 | 0.28 | 0.1 |
| **Soil Available Iron** | 8.8 | 6.8 | 4.3 | 1.7 | 2.6 | 2 | 7.3 | 2.7 | 2.3 | 3 |
| **Soil Available Manganese** | 1 | 0.6 | 0.8 | 4.1 | 1.3 | 2.3 | 1.2 | 0.5 | 0.3 | 0.8 |
| **Total Water-Soluble Salt** | 2.4 | 2.1 | 2.3 | 1.2 | 2.6 | 2 | 2.2 | 2.8 | 1.6 | 4.4 |
| **Soil Total Phosphorus** | 351 | 191 | 347 | 638 | 250 | 262 | 187 | 156 | 6720 | 7230 |
| **Soil Available Sulphur** | 24.6 | 5.29 | 8.19 | 1.93 | 22.6 | 66.2 | 33.1 | 32.3 | 9.58 | 13.8 |
| **Soil Total Nitrogen** | 0.069 | 0.048 | 0.063 | 0.087 | 0.053 | 0.064 | 0.057 | 0.047 | 0.222 | 0.214 |
| **Soil Nitrite Nitrogen** | 1.48 | 0.7 | 0.48 | 0.45 | 0.44 | 0.81 | 0.39 | 0.34 | 0.58 | 0.59 |
| **Soil Nitrate Nitrogen** | 60.6 | 10.3 | 29.9 | 9.32 | 13.6 | 14.2 | 42 | 16.1 | 8 | 8.25 |
| **Soil Ammonium Nitrogen** | 2.24 | 0.9 | 0.8 | 12.42 | 0.74 | 10.51 | 2.77 | 0.36 | 0.66 | 1.21 |
| **Soil Ammonia Nitrogen** | 0.45 | 4.58 | 4.92 | 3.42 | 1.75 | 7.71 | 6.36 | 10.6 | 5.54 | 4.65 |

**Table S2 Continued**

|  | **YX.3** | **YX.4** | **YX.5** | **YX.6** | **ZX.1** | **N5.11** | **N5.21** | **N5.31** | **N7.11** | **N7.21** |
| --- | --- | --- | --- | --- | --- | --- | --- | --- | --- | --- |
| **pH** | 8.39 | 8.85 | 9.3 | 8.38 | 9.32 | 9.67 | 9.63 | 9.56 | 8.81 | 9.57 |
| **Soil Available Boron** | 0.024 | 0.03 | 0.134 | 1.22 | 0.063 | 0.014 | 0.006 | 0.01 | 0.004 | 0.035 |
| **Organic Matter** | 28.8 | 22 | 5.45 | 15.3 | 12.7 | 3.84 | 3.69 | 4.95 | 6.55 | 3.6 |
| **Organic Carbon** | 1.44 | 1.39 | 0.51 | 0.79 | 0.93 | 0.3 | 0.28 | 0.27 | 0.18 | 0.26 |
| **Soil Available Phosphorus** | 44.6 | 26.1 | 10.6 | 15 | 60.5 | 21.1 | 12.4 | 13 | 28.5 | 21.6 |
| **Soil Exchangeable Calcium** | 25.9 | 86 | 67.4 | 98 | 67.4 | 50.1 | 79.2 | 51.5 | 10.6 | 78.8 |
| **Soil Available Kalium** | 106 | 32.9 | 21.6 | 79.3 | 16 | 33.6 | 20.6 | 38.4 | 27.5 | 82.1 |
| **Kalium** | 3300 | 321 | 190 | 1000 | 179 | 799 | 298 | 1000 | 8533 | 401 |
| **Calcium** | 0.96 | 254 | 303 | 292 | 295 | 230 | 410 | 268 | 0.72 | 289 |
| **Soil Available Zinc** | 4.29 | 8.05 | 7.6 | 7.99 | 10.9 | 11 | 4.32 | 4.48 | 4.14 | 6.61 |
| **Soil Available Copper** | 0.31 | 0.31 | 0.09 | 0.17 | 0.11 | 0.15 | 0.08 | 0.06 | 0.05 | 0.025 |
| **Soil Available Iron** | 0.9 | 9.1 | 1.9 | 2.5 | 3.3 | 7 | 5 | 6.1 | 1.2 | 2.4 |
| **Soil Available Manganese** | 7.5 | 0.9 | 0.4 | 0.7 | 0.6 | 0.2 | 0.2 | 0.3 | 2.6 | 1.8 |
| **Total Water-Soluble Salt** | 2.4 | 2 | 6.3 | 10.5 | 2.5 | 2.6 | 5.5 | 2.6 | 2.6 | 2.8 |
| **Soil Total Phosphorus** | 1237 | 1110 | 165 | 529 | 759 | 181 | 172 | 181 | 771 | 166 |
| **Soil Available Sulphur** | 23.6 | 23.3 | 268 | 3340 | 20.1 | 6.2 | 2.31 | 3.56 | 6.52 | 3.14 |
| **Soil Total Nitrogen** | 0.156 | 0.079 | 0.029 | 0.066 | 0.083 | 0.038 | 0.018 | 0.023 | 0.062 | 0.02 |
| **Soil Nitrite Nitrogen** | 0.89 | 0.45 | 15 | 1.74 | 1.26 | 0.93 | 0.79 | 0.33 | 0.77 | 0.69 |
| **Soil Nitrate Nitrogen** | 9.64 | 8.05 | 16.3 | 50.3 | 11 | 9.87 | 7.83 | 10.3 | 7.7 | 8.37 |
| **Soil Ammonium Nitrogen** | 4.44 | 0.5 | 28.66 | 3.81 | 0.92 | 0.05 | 0.1 | 0.15 | 1.25 | 0.1 |
| **Soil Ammonia Nitrogen** | 6.54 | 6.82 | 19.2 | 9.81 | 13.1 | 4.71 | 3.56 | 4.46 | 7.25 | 2.1 |

**Table S2 Continued**

|  | **N7.31** | **N7.41** | **N7.51** | **YX.11** | **YX.21** | **YX.31** | **YX.41** | **YX.51** | **YX.61** | **ZX.11** |
| --- | --- | --- | --- | --- | --- | --- | --- | --- | --- | --- |
| **pH** | 9.62 | 9.59 | 9.6 | 8.95 | 9 | 9.04 | 9.53 | 9.61 | 9.15 | 9.66 |
| **Soil Available Boron** | 0.057 | 0.093 | 0.028 | 0.011 | 0.016 | 0.022 | 0.018 | 0.023 | 0.103 | 0.039 |
| **Organic Matter** | 10.2 | 3.73 | 3.67 | 31.6 | 22.4 | 15.9 | 8.28 | 2.1 | 4.7 | 5.47 |
| **Organic Carbon** | 1.95 | 0.33 | 0.27 | 1.68 | 1.56 | 1.08 | 0.9 | 0.3 | 0.49 | 0.43 |
| **Soil Available Phosphorus** | 22.7 | 14.6 | 16.5 | 134 | 102 | 17.5 | 27.3 | 10.8 | 15.8 | 34.6 |
| **Soil Exchangeable Calcium** | 60.7 | 104 | 87.6 | 32.9 | 40.6 | 24.4 | 73.5 | 83.4 | 93.3 | 47.3 |
| **Soil Available Kalium** | 58 | 25.1 | 21.8 | 33.4 | 12.2 | 86.1 | 94 | 10 | 29.6 | 14 |
| **Kalium** | 3500 | 228 | 130 | 332 | 130 | 2000 | 1833 | 92.6 | 292 | 92.4 |
| **Calcium** | 1.98 | 259 | 337 | 300 | 80.8 | 228 | 276 | 241 | 312 | 306 |
| **Soil Available Zinc** | 18.1 | 0.89 | 0.12 | 6.69 | 6.12 | 5.49 | 4.68 | 2.44 | 5.89 | 7.02 |
| **Soil Available Copper** | 6.59 | 0.65 | 0.22 | 0.011 | 0 | 0.09 | 0.05 | 0.05 | 0 | 0.07 |
| **Soil Available Iron** | 21.5 | 9.5 | 1.6 | 1.7 | 1.9 | 0.3 | 6.7 | 1.4 | 3 | 1.3 |
| **Soil Available Manganese** | 2.5 | 0.5 | 0.2 | 0.2 | 0.3 | 1.7 | 0.3 | 0.2 | 0.2 | 0.2 |
| **Total Water-Soluble Salt** | 2.4 | 1.6 | 1.6 | 3.5 | 1.6 | 2.1 | 2.4 | 1.6 | 1.4 | 1.9 |
| **Soil Total Phosphorus** | 158 | 168 | 145 | 7430 | 4300 | 1313 | 1076 | 172 | 328 | 346 |
| **Soil Available Sulphur** | 11.4 | 25.6 | 13.8 | 1.87 | 10.5 | 29.6 | 11.1 | 12.8 | 104 | 16.9 |
| **Soil Total Nitrogen** | 0.026 | 0.021 | 0.024 | 0.177 | 0.121 | 0.076 | 0.034 | 0.016 | 0.022 | 0.036 |
| **Soil Nitrite Nitrogen** | 0.39 | 0.51 | 0.75 | 0.45 | 0.76 | 0.57 | 0.73 | 0.68 | 0.48 | 0.42 |
| **Soil Nitrate Nitrogen** | 8.31 | 13.2 | 11.7 | 8.08 | 8.13 | 8.28 | 8.23 | 10 | 15.8 | 11.1 |
| **Soil Ammonium Nitrogen** | 5.09 | 6.72 | 0.15 | 0.15 | 0.1 | 2.17 | 0.18 | 0.1 | 0.1 | 0.1 |
| **Soil Ammonia Nitrogen** | 4.52 | 3.95 | 2 | 4.93 | 3.08 | 6.76 | 4.14 | 4.51 | 1.74 | 4.46 |

**Table S2 Continued**

|  | **N5.01** | **N5.02** | **N5.03** | **N5.04** | **N7.01** | **N7.02** | **N7.03** | **N7.04** | **YX.01** | **YX.02** | **ZX.0** |
| --- | --- | --- | --- | --- | --- | --- | --- | --- | --- | --- | --- |
| **pH** | 9.61 | 9.57 | 9.59 | 9.67 | 9.62 | 9.65 | 9.71 | 9.5 | 9.71 | 9.7 | 9.68 |
| **Soil Available Boron** | 0.013 | 0.012 | 0.013 | 0.013 | 0.028 | 0.028 | 0.029 | 0.027 | 0.037 | 0.038 | 0.028 |
| **Organic Matter** | 8.01 | 8.05 | 8.02 | 7.98 | 2.89 | 2.89 | 2.95 | 2.83 | 4.11 | 4.15 | 74.2 |
| **Organic Carbon** | 0.61 | 0.6 | 0.6 | 0.62 | 0.48 | 0.48 | 0.49 | 0.48 | 0.38 | 0.37 | 4.53 |
| **Soil Available Phosphorus** | 17.4 | 12.1 | 12.8 | 12.4 | 17.8 | 17.8 | 18.1 | 17.4 | 15.4 | 15.1 | 295 |
| **Soil Exchangeable Calcium** | 46.3 | 46.1 | 45.4 | 47.4 | 109 | 108 | 110 | 110 | 30.8 | 31.1 | 37.7 |
| **Soil Available Kalium** | 21.6 | 21.7 | 21.7 | 21.3 | 13.8 | 13.8 | 13.8 | 13.7 | 17.8 | 17.7 | 21.1 |
| **Kalium** | 706 | 709 | 704 | 704 | 76.5 | 76.4 | 76.3 | 76.8 | 246 | 251 | 239 |
| **Calcium** | 285 | 281 | 286 | 287 | 361 | 359 | 363 | 361 | 245 | 248 | 224 |
| **Soil Available Zinc** | 5.51 | 5.57 | 5.63 | 5.33 | 5.18 | 5.17 | 5.2 | 5.16 | 5.27 | 5.1 | 6.03 |
| **Soil Available Copper** | 0.06 | 0.06 | 0.07 | 0.06 | 0.006 | 0 | 0.0166 | 0 | 0.04 | 0.04 | 0.13 |
| **Soil Available Iron** | 7.9 | 7.9 | 7.8 | 7.9 | 2.9 | 2.9 | 2.9 | 3 | 2.1 | 2.2 | 1.2 |
| **Soil Available Manganese** | 0.2 | 0.2 | 0.2 | 0.2 | 0.2 | 0.2 | 0.2 | 0.2 | 0.2 | 0.2 | 0.4 |
| **Total Water-Soluble Salt** | 3.6 | 3.6 | 3.9 | 3.2 | 2.6 | 2.9 | 2.5 | 2.3 | 2.4 | 2.3 | 1.4 |
| **Soil Total Phosphorus** | 191 | 195 | 193 | 184 | 156 | 155 | 157 | 155 | 496 | 496 | 54500 |
| **Soil Available Sulphur** | 9.61 | 9.68 | 9.47 | 9.68 | 74.5 | 74.3 | 74.1 | 75.1 | 15.3 | 15.3 | 13.8 |
| **Soil Total Nitrogen** | 0.018 | 0.019 | 0.016 | 0.018 | 0.017 | 0.017 | 0.017 | 0.018 | 0.024 | 0.025 | 0.536 |
| **Soil Nitrite Nitrogen** | 0.48 | 0.53 | 0.42 | 0.49 | 0.78 | 0.71 | 0.8 | 0.83 | 0.98 | 1.03 | 0.51 |
| **Soil Nitrate Nitrogen** | 8.77 | 8.9 | 8.86 | 8.54 | 10.8 | 10.7 | 10.9 | 10.9 | 9.96 | 9.77 | 11.2 |
| **Soil Ammonium Nitrogen** | 3.06 | 3.06 | 3.11 | 3.01 | 0.27 | 0.31 | 0.26 | 0.26 | 0.21 | 0.25 | 3.73 |
| **Soil Ammonia Nitrogen** | 0.2 | 0.2 | 0.2 | 0.21 | 0.17 | 0.16 | 0.18 | 0.18 | 0.3 | 0.31 | 0.26 |

Note: N5.x and N7.x: BSC samples from Nansha Archipelagos; N5.x1 and N7.x1: BSC subsurface soil samples from Nansha Archipelagos; YX.x and ZX.x: BSC samples from Xisha Archipelagos; YX.x1 and ZX.x1: BSC subsurface soil samples from Xisha Archipelagos; N5.0x and N7.0x: bare soil samples from Nansha Archipelagos; YX.0x and ZX.0x: bare soil samples from Xisha Archipelagos; Biological soil crust (BSC); Soil characteristics units: Soil Available Boron (mg kg^-1^), Organic Matter (g kg^-1^), Organic Carbon (%), Soil Available Phosphorus (mg kg^-1^), Soil Exchangeable Calcium (cmol(1/2Ca^2+^) kg^-1^), Soil Available Kalium (mg kg^-1^), Kalium (mg kg^-1^), Calcium (g kg^-1^), Soil Available Zinc (mg kg^-1^), Soil Available Copper (mg kg^-1^), Soil Available Iron (mg kg^-1^), Soil Available Manganese (mg kg^-1^), Total Water Soluble Salt (g kg^-1^), Soil Total Phosphorus (mg kg^-1^), Soil Available Sulphur (mg kg^-1^), Soil Total Nitrogen (%), Soil Nitrite Nitrogen (mg kg^-1^), Soil Nitrate Nitrogen (mg kg^-1^), Soil Ammonium Nitrogen (mg kg^-1^), Soil Ammonia Nitrogen (mg kg^-1^).

**Table S3 The richness and diversity of bacterial, fungi and archaea communities in the biocrust samples.**

|  | **Bacteria** | | | **Fungi** | | | **Archaea** | | |
| --- | --- | --- | --- | --- | --- | --- | --- | --- | --- |
| **Sample** | **chao1** | **observed _species** | **shannon** | **chao1** | **observed _species** | **shannon** | **chao1** | **observed _species** | **shannon** |
| **N5.01** | **4232.554** | **2133** | **5.176** | **109.273** | **69** | **1.386** | **44.413** | **35** | **3.839** |
| **N5.02** | **1768.917** | **1269** | **5.207** | **35.740** | **24** | **1.116** | **27.250** | **22** | **3.244** |
| **N5.03** | **1891.854** | **1378** | **5.321** | **36.694** | **32** | **1.615** | **22.087** | **13** | **2.541** |
| **N5.04** | **1987.212** | **1420** | **5.364** | **43.758** | **32** | **1.896** | **17.382** | **14** | **2.614** |
| **N7.01** | **5071.753** | **3062** | **8.633** | **NA** | **NA** | **NA** | **36.616** | **21** | **2.741** |
| **N7.02** | **2214.750** | **1613** | **8.361** | **49.616** | **39** | **1.976** | **17.108** | **16** | **2.501** |
| **N7.03** | **2760.278** | **2095** | **8.210** | **42.131** | **37** | **2.178** | **17.703** | **13** | **2.193** |
| **N7.04** | **1622.872** | **1016** | **7.747** | **31.015** | **28** | **1.684** | **8.850** | **8** | **2.063** |
| **YX.01** | **6822.913** | **4836** | **10.262** | **446.748** | **308** | **3.409** | **67.267** | **40** | **4.155** |
| **YX.02** | **4168.394** | **3347** | **8.620** | **41.106** | **36** | **2.021** | **44.137** | **29** | **3.831** |
| **ZX.01** | **8384.583** | **5851** | **10.632** | **551.691** | **391** | **3.874** | **26.307** | **20** | **3.051** |
| **N5.1** | **4188.463** | **2328** | **6.161** | **366.372** | **207** | **2.109** | **NA** | **NA** | **NA** |
| **N5.2** | **5824.761** | **3300** | **7.286** | **331.743** | **210** | **4.326** | **34.938** | **18** | **1.680** |
| **N5.3** | **5760.692** | **3614** | **8.678** | **452.680** | **289** | **3.268** | **15.665** | **7** | **0.309** |
| **N7.1** | **5441.181** | **3200** | **7.459** | **343.114** | **196** | **1.379** | **8.175** | **5** | **0.206** |
| **N7.2** | **5599.858** | **3228** | **8.145** | **460.839** | **272** | **3.293** | **6.733** | **4** | **0.124** |
| **N7.3** | **3453.135** | **2578** | **8.319** | **686.725** | **443** | **4.267** | **8.050** | **4** | **0.135** |
| **N7.4** | **4520.401** | **2607** | **7.625** | **500.076** | **306** | **3.328** | **10.313** | **5** | **0.164** |
| **N7.5** | **5860.189** | **3329** | **8.621** | **300.886** | **168** | **1.746** | **37.709** | **25** | **3.210** |
| **YX.1** | **7219.623** | **4486** | **9.070** | **486.686** | **385** | **5.270** | **12.850** | **10** | **1.768** |
| **YX.2** | **6520.649** | **3738** | **6.919** | **500.256** | **333** | **3.433** | **12.144** | **11** | **2.716** |
| **YX.3** | **6893.506** | **4490** | **9.377** | **625.047** | **411** | **5.150** | **8.408** | **6** | **1.265** |
| **YX.4** | **5450.221** | **3251** | **7.678** | **660.206** | **451** | **4.425** | **23.980** | **16** | **2.372** |
| **YX.5** | **4769.148** | **2531** | **6.784** | **336.723** | **223** | **3.106** | **NA** | **NA** | **NA** |
| **YX.6** | **6144.721** | **3668** | **8.298** | **413.768** | **258** | **2.246** | **111.911** | **71** | **5.423** |
| **ZX.1** | **5653.906** | **2834** | **6.664** | **330.222** | **205** | **1.529** | **38.802** | **16** | **0.969** |
| **N5.11** | **5694.585** | **3592** | **8.427** | **361.624** | **230** | **2.220** | **60.932** | **39** | **3.818** |
| **N5.21** | **2722.983** | **1820** | **6.163** | **378.604** | **203** | **0.829** | **30.304** | **21** | **2.688** |
| **N5.31** | **3637.498** | **2668** | **8.575** | **158.977** | **115** | **3.696** | **61.474** | **43** | **4.128** |
| **N7.11** | **4052.809** | **3099** | **9.158** | **496.868** | **355** | **4.029** | **39.479** | **34** | **3.887** |
| **N7.21** | **4750.815** | **3287** | **8.901** | **532.359** | **314** | **3.900** | **42.726** | **36** | **4.025** |
| **N7.31** | **6378.684** | **3833** | **8.772** | **371.921** | **224** | **2.255** | **67.792** | **41** | **4.423** |
| **N7.41** | **6091.419** | **3674** | **9.028** | **593.924** | **384** | **4.952** | **26.542** | **15** | **1.941** |
| **N7.51** | **5954.993** | **3591** | **8.344** | **476.209** | **297** | **3.175** | **53.115** | **36** | **3.656** |
| **YX.11** | **8402.256** | **5555** | **10.182** | **211.819** | **146** | **4.617** | **25.632** | **20** | **3.225** |
| **YX.21** | **8737.545** | **6001** | **10.538** | **311.333** | **238** | **5.433** | **31.215** | **24** | **3.634** |
| **YX.31** | **8679.827** | **5780** | **10.368** | **588.386** | **469** | **5.961** | **28.025** | **22** | **2.742** |
| **YX.41** | **4397.873** | **2743** | **6.631** | **NA** | **NA** | **NA** | **33.296** | **25** | **3.647** |
| **YX.51** | **5894.934** | **3555** | **8.083** | **328.331** | **276** | **4.796** | **60.488** | **37** | **3.061** |
| **YX.61** | **7490.508** | **4881** | **9.812** | **110.074** | **75** | **2.080** | **103.650** | **57** | **4.674** |
| **ZX.11** | **7195.333** | **4705** | **9.656** | **NA** | **NA** | **NA** | **104.202** | **60** | **5.012** |

Note: N5.x and N7.x: BSC samples from Nansha Archipelagos; N5.x1 and N7.x1: BSC subsurface soil samples from Nansha Archipelagos; YX.x and ZX.x: BSC samples from Xisha Archipelagos; YX.x1 and ZX.x1: BSC subsurface soil samples from Xisha Archipelagos; N5.0x and N7.0x: bare soil samples from Nansha Archipelagos; YX.0x and ZX.0x: bare soil samples from Xisha Archipelagos

**Table S4 Soil enzyme activities and Chl a contents from South China Sea**

|  | S-CAT | S-FDA | S-ALPT | S-AKP | S-UE | S-β-GC | S-LPS | Chl a |
| --- | --- | --- | --- | --- | --- | --- | --- | --- |
| N5.1 | 52 | 54.33 | 126.99 | 16.01 | 813.96 | 1.28 | 40.77 | 27.34 |
| N5.2 | 27.19 | 37.06 | 295.03 | 16.96 | 715.63 | 1 | 38.19 | 17.9 |
| N5.3 | 32.32 | 41.26 | 292.23 | 16.86 | 749.92 | 0.97 | 39.78 | 16.19 |
| N7.1 | 39.48 | 49.27 | 71.76 | 16.89 | 851.59 | 2.32 | 37.53 | 9.21 |
| N7.2 | 45.9 | 46.01 | 173.49 | 16.85 | 817.3 | 1.47 | 35.35 | 20.12 |
| N7.3 | 55.47 | 41.07 | 100.77 | 17.25 | 861.31 | 1.69 | 35.15 | 34.35 |
| N7.4 | 43.32 | 52.87 | 226.97 | 16.52 | 811.23 | 2.28 | 32.77 | 18.11 |
| N7.5 | 49.3 | 46.74 | 186.42 | 16.94 | 710.47 | 1.55 | 38.59 | 10.17 |
| YX.1 | 49.31 | 54.37 | 39.36 | 14.49 | 805.77 | 1.96 | 30.66 | 23.42 |
| YX.2 | 38.8 | 29.35 | 27.48 | 9.64 | 816.39 | 0.94 | 35.15 | 13.93 |
| YX.3 | 44.69 | 46.23 | 2.42 | 19.44 | 893.17 | 1.19 | 37 | 10.65 |
| YX.4 | 37.61 | 55.07 | 5.45 | 17.66 | 844.31 | 1.54 | 39.84 | 10.73 |
| YX.5 | 18.29 | 23.85 | 141.68 | 7.67 | 204.25 | 0.75 | 37.73 | 20.08 |
| YX.6 | 20.16 | 50.8 | 84.34 | 19.05 | 620.64 | 0.44 | 37.53 | 27.99 |
| ZX.1 | 31.76 | 32.33 | 24.56 | 16.2 | 596.66 | 2.2 | 34.82 | 7.01 |
| N5.11 | 5.34 | 2.37 | 312.74 | 0.79 | 57.36 | 0.03 | 44.01 | 0.05 |
| N5.21 | 5.25 | 2.84 | 281.04 | 0.87 | 37.33 | 0.14 | 34.16 | 0.05 |
| N5.31 | 6.69 | 2.88 | 310.88 | 0.84 | 55.54 | 0.02 | 39.25 | 0.01 |
| N7.11 | 25.36 | 2.85 | 1.61 | 2.27 | 246.13 | 0.16 | 33.57 | 0.03 |
| N7.21 | 10.11 | 2.94 | 308.2 | 0.84 | 78 | 0.03 | 32.18 | 0.08 |
| N7.31 | 17.8 | 2.89 | 202.39 | 0.37 | 44.31 | 0.06 | 32.64 | 0.01 |
| N7.41 | 10.77 | 3.31 | 222.66 | 1.94 | 79.82 | 0.08 | 33.9 | 0.02 |
| N7.51 | 6.59 | 3.01 | 271.49 | 1.01 | 64.64 | 0.09 | 34.43 | 0.03 |
| YX.11 | 19.46 | 5.34 | 12.1 | 4.26 | 365.71 | 0.04 | 32.44 | 0.02 |
| YX.21 | 8.03 | 4.39 | 4.06 | 2.5 | 583.61 | 0.05 | 32.18 | 0.01 |
| YX.31 | 34.3 | 17.66 | 2.66 | 10.94 | 817.6 | 0.73 | 34.95 | 0.04 |
| YX.41 | 5.62 | 3.36 | 27.94 | 0.58 | 128.07 | 0.03 | 37.14 | 0.01 |
| YX.51 | 4.13 | 2.29 | 126.41 | 0.38 | 48.56 | 0.08 | 35.22 | 0.02 |
| YX.61 | 6 | 4.07 | 24.1 | 0.55 | 79.21 | 0.1 | 37.47 | 0.29 |
| ZX.11 | 7.39 | 2.93 | 140.51 | 0.64 | 80.12 | 0.1 | 35.09 | 0.05 |
| N5.01 | 7.22 | 4.31 | 75.6 | 1.44 | 75.27 | 0.1 | 33.17 | 0 |
| N5.02 | 7.1 | 4.18 | 93.67 | 1.69 | 77.09 | 0.13 | 35.15 | 0 |
| N5.03 | 7.03 | 4.62 | 66.05 | 1.25 | 78 | 0.1 | 33.17 | 0 |
| N5.04 | 7.52 | 4.12 | 67.1 | 1.37 | 70.71 | 0.07 | 31.19 | 0.01 |
| N7.01 | 5.72 | 2.81 | 157.06 | 0.08 | 38.85 | 0.12 | 33.3 | 0.04 |
| N7.02 | 5.52 | 2.82 | 184.56 | 0.06 | 43.7 | 0.22 | 34.36 | 0.04 |
| N7.03 | 5.95 | 2.87 | 162.88 | 0.09 | 35.51 | 0.08 | 30 | 0 |
| N7.04 | 5.69 | 2.73 | 123.73 | 0.09 | 37.33 | 0.05 | 35.55 | 0.07 |
| YX.01 | 6.13 | 3.1 | 116.86 | 1.29 | 163.58 | 0.02 | 38.26 | 0.05 |
| YX.02 | 6.4 | 2.98 | 109.98 | 2.13 | 179.62 | 0.07 | 38.03 | 0.05 |
| ZX.0 | 50.42 | 30.93 | 83.53 | 18.47 | 548.41 | 3.83 | 35.09 | 0.05 |

Note: N5.x and N7.x: BSC samples from Nansha Archipelagos; N5.x1 and N7.x1: BSC subsurface soil samples from Nansha Archipelagos; YX.x and ZX.x: BSC samples from Xisha Archipelagos; YX.x1 and ZX.x1: BSC subsurface soil samples from Xisha Archipelagos; N5.0x and N7.0x: bare soil samples from Nansha Archipelagos; YX.0x and ZX.0x: bare soil samples from Xisha Archipelagos; Abbreviations: chlorophyll a (Chl a), soil β-glucosidase activity (S-β-GC), soil lipase activity (S-LPS), soil FDA hydrolase activity (S-FDA), soil alkaline protease activity (S-ALPT), soil urease activity (S-UE), soil alkaline phosphatase activity (S-AKP), soil catalase activity (S-CAT). Units: chlorophyll a (μg g^-1^), soil β-glucosidase activity (μmol d^-1^ g^-1^), soil lipase activity (μmol d^-1^ g^-1^), soil FDA hydrolase activity (μmol d^-1^ g^-1^), soil alkaline protease activity (μmol d^-1^ g^-1^), soil urease activity (μg d^-1^ g^-1^), soil alkaline phosphatase activity (μmol d^-1^ g^-1^), soil catalase activity (μmol d^-1^ g^-1^).

**Table S5 PERMANOVA analysis of microbial community composition among different soil types**

| Bacterial community | | | |
| --- | --- | --- | --- |
| Type | F. Model | R^2^ | *p*. value |
| BSCs vs BSC_sub vs BS | 3.848 | 0.168 | 0.001 |
| BS vs BSCs | 4.850 | 0.168 | 0.001 |
| BS vs BSC_sub | 3.291 | 0.121 | 0.001 |
| BSCs vs BSC_sub | 3.505 | 0.111 | 0.001 |
| BS(NS) vs BS(XS) | 3.313 | 0.269 | 0.025 |
| BSCs(NS) vs BSCs(XS) | 2.279 | 0.149 | 0.002 |
| BSC_sub(NS) vs BSC_sub(XS) | 2.140 | 0.141 | 0.001 |
| Archaeal community | | | |
| Type | F. Model | R^2^ | *p*. value |
| BSCs vs BSC_sub vs BS | 5.329 | 0.228 | 0.001 |
| BS vs BSCs | 8.760 | 0.285 | 0.001 |
| BS vs BSC_sub | 2.639 | 0.099 | 0.010 |
| BSCs vs BSC_sub | 5.598 | 0.177 | 0.001 |
| BS(NS) vs BS(XS) | 4.059 | 0.311 | 0.006 |
| BSCs(NS) vs BSCs(XS) | 1.685 | 0.133 | 0.116 |
| BSC_sub(NS) vs BSC_sub(XS) | 1.346 | 0.094 | 0.200 |
| Fungal community | | | |
| Type | F. Model | R^2^ | *p*. value |
| BSCs vs BSC_sub vs BS | 2.110 | 0.108 | 0.001 |
| BS vs BSCs | 3.096 | 0.119 | 0.001 |
| BS vs BSC_sub | 1.275 | 0.057 | 0.037 |
| BSCs vs BSC_sub | 2.019 | 0.072 | 0.007 |
| BS(NS) vs BS(XS) | 1.413 | 0.150 | 0.028 |
| BSCs(NS) vs BSCs(XS) | 1.174 | 0.083 | 0.250 |
| BSC_sub(NS) vs BSC_sub(XS) | 1.484 | 0.119 | 0.007 |

Abbreviations: Nansha Archipelagos (NS), Xisha Archipelagos (XS), bare soil (BS), BSC subsurface soil (BSC_sub), and biological soil crusts (BSCs)

**Table S6 Correlation between soil parameters and NMDS axes**

| **Bacteria** | **NMDS1** | **NMDS2** | **r^2^** | **Pr(>r)** |
| --- | --- | --- | --- | --- |
| pH | 0.93362 | -0.35825 | 0.2929 | 0.002 |
| B | -0.97395 | 0.22676 | 0.1507 | 0.031 |
| S | -0.98731 | 0.15879 | 0.1469 | 0.041 |
| NO_3_-N | -0.99995 | -0.01021 | 0.134 | 0.045 |
| NH_3_-N | -0.62139 | 0.7835 | 0.3638 | 0.001 |
| **Fungi** | **NMDS1** | **NMDS2** | **r^2^** | **Pr(>r)** |
| pH | -0.65938 | -0.75181 | 0.1908 | 0.024 |
| Ca | -0.52267 | -0.85253 | 0.2051 | 0.013 |
| Mn | 0.51153 | 0.85927 | 0.2086 | 0.015 |
| **Archaea** | **NMDS1** | **NMDS2** | **r^2^** | **Pr(>r)** |
| Ca | 0.64332 | 0.7656 | 0.1557 | 0.048 |

Abbreviations: Soil Available Boron (B); Soil Available Sulphur (S); Soil Nitrate Nitrogen (NO_3_-N); Soil Ammonia Nitrogen (NH_3_-N); Soil Available Manganese (Mn); Calcium (Ca).

**Table S7 OTUs classification information for specialists in BSCs**

| OTU | taxonomy |
| --- | --- |
| bacteria_OTU218 | k__Bacteria; p__Bacteroidetes; c__Cytophagia; o__Cytophagales; f__Cytophagaceae; g__; s__ |
| bacteria_OTU732 | k__Bacteria; p__Bacteroidetes; c__Cytophagia; o__Cytophagales; f__Cytophagaceae; g__Hymenobacter; s__ |
| bacteria_OTU37 | k__Bacteria; p__Bacteroidetes; c__Cytophagia; o__Cytophagales; f__Flammeovirgaceae; g__; s__ |
| bacteria_OTU450 | k__Bacteria; p__Bacteroidetes; c__Cytophagia; o__Cytophagales; f__Flammeovirgaceae; g__; s__ |
| bacteria_OTU1347 | k__Bacteria; p__Bacteroidetes; c__Flavobacteriia; o__Flavobacteriales; f__Cryomorphaceae; g__Cryomorpha; s__ |
| bacteria_OTU331 | k__Bacteria; p__Chlorobi; c__OPB56; o__; f__; g__; s__ |
| bacteria_OTU187 | k__Bacteria; p__Chloroflexi; c__Anaerolineae; o__SBR1031; f__A4b; g__; s__ |
| bacteria_OTU751 | k__Bacteria; p__Chloroflexi; c__Anaerolineae; o__SBR1031; f__A4b; g__; s__ |
| bacteria_OTU2238 | k__Bacteria; p__Chloroflexi; c__Chloroflexi; o__Chloroflexales; f__Chloroflexaceae; g__Chloronema; s__ |
| bacteria_OTU52 | k__Bacteria; p__Chloroflexi; c__Chloroflexi; o__Chloroflexales; f__Chloroflexaceae; g__Chloronema; s__ |
| bacteria_OTU115 | k__Bacteria; p__Cyanobacteria |
| bacteria_OTU366 | k__Bacteria; p__Cyanobacteria |
| bacteria_OTU44414 | k__Bacteria; p__Cyanobacteria; c__; o__; f__; g__; s__ |
| bacteria_OTU771 | k__Bacteria; p__Cyanobacteria; c__; o__; f__; g__; s__ |
| bacteria_OTU87 | k__Bacteria; p__Cyanobacteria; c__; o__; f__; g__; s__ |
| bacteria_OTU228 | k__Bacteria; p__Cyanobacteria; c__Nostocophycideae; o__Nostocales; f__Nostocaceae |
| bacteria_OTU15980 | k__Bacteria; p__Cyanobacteria; c__Nostocophycideae; o__Nostocales; f__Scytonemataceae; g__Scytonema; s__ |
| bacteria_OTU29488 | k__Bacteria; p__Cyanobacteria; c__Nostocophycideae; o__Nostocales; f__Scytonemataceae; g__Scytonema; s__ |
| bacteria_OTU899 | k__Bacteria; p__Cyanobacteria; c__Oscillatoriophycideae; o__Chroococcales; f__Cyanobacteriaceae |
| bacteria_OTU18766 | k__Bacteria; p__Cyanobacteria; c__Oscillatoriophycideae; o__Oscillatoriales; f__Phormidiaceae; g__Phormidium |
| bacteria_OTU146 | k__Bacteria; p__Cyanobacteria; c__Oscillatoriophycideae; o__Oscillatoriales; f__Phormidiaceae; g__Phormidium; s__ |
| bacteria_OTU172 | k__Bacteria; p__Cyanobacteria; c__Synechococcophycideae; o__Pseudanabaenales; f__Pseudanabaenaceae; g__; s__ |
| bacteria_OTU32472 | k__Bacteria; p__Cyanobacteria; c__Synechococcophycideae; o__Pseudanabaenales; f__Pseudanabaenaceae; g__; s__ |
| bacteria_OTU314 | k__Bacteria; p__Cyanobacteria; c__Synechococcophycideae; o__Pseudanabaenales; f__Pseudanabaenaceae; g__Halomicronema; s__ |

**Table S7 Continued**

| OTU | taxonomy |
| --- | --- |
| bacteria_OTU86 | k__Bacteria; p__Cyanobacteria; c__Synechococcophycideae; o__Pseudanabaenales; f__Pseudanabaenaceae; g__Halomicronema; s__ |
| bacteria_OTU1257 | k__Bacteria; p__Cyanobacteria; c__Synechococcophycideae; o__Pseudanabaenales; f__Pseudanabaenaceae; g__Leptolyngbya; s__ |
| bacteria_OTU402 | k__Bacteria; p__Cyanobacteria; c__Synechococcophycideae; o__Pseudanabaenales; f__Pseudanabaenaceae; g__Leptolyngbya; s__frigida |
| bacteria_OTU1228 | k__Bacteria; p__Proteobacteria; c__Alphaproteobacteria |
| bacteria_OTU36 | k__Bacteria; p__Proteobacteria; c__Alphaproteobacteria |
| fungi_OTU145 | k__Fungi; p__Ascomycota; c__Dothideomycetes; o__Pleosporales; f__unidentified; g__unidentified |
| fungi_OTU25 | k__Fungi; p__Ascomycota; c__Dothideomycetes; o__Pleosporales; f__unidentified; g__unidentified |
| fungi_OTU34 | k__Fungi; p__Ascomycota; c__Lecanoromycetes; o__Lecanorales; f__Lecanorales_fam_Incertae_sedis; g__Leprocaulon |
| fungi_OTU26 | k__Fungi; p__Ascomycota; c__Orbiliomycetes; o__Orbiliales; f__Orbiliaceae; g__unidentified |
| fungi_OTU135 | k__Fungi; p__Ascomycota; c__Pezizomycetes; o__Pezizales; f__Helvellaceae; g__Helvella |
| fungi_OTU69 | k__Fungi; p__Ascomycota; c__Pezizomycotina_cls_Incertae_sedis; o__Pezizomycotina_ord_Incertae_sedis; f__Pezizomycotina_fam_Incertae_sedis; g__Knufia |
| fungi_OTU86 | k__Fungi; p__Ascomycota; c__Pezizomycotina_cls_Incertae_sedis; o__Pezizomycotina_ord_Incertae_sedis; f__Pezizomycotina_fam_Incertae_sedis; g__Ochroconis |
| fungi_OTU35 | k__Fungi; p__Ascomycota; c__Sordariomycetes; o__Ophiostomatales; f__unidentified; g__unidentified |
| fungi_OTU380 | k__Fungi; p__Ascomycota; c__Sordariomycetes; o__Sordariomycetidae_ord_Incertae_sedis; f__Glomerellaceae; g__Colletotrichum |
| fungi_OTU38 | k__Fungi; p__Ascomycota; c__unidentified; o__unidentified; f__unidentified; g__unidentified |
| fungi_OTU57 | k__Fungi; p__Basidiomycota; c__Agaricomycetes; o__Agaricales; f__Psathyrellaceae; g__Coprinopsis |
| fungi_OTU194 | k__Fungi; p__Basidiomycota; c__Agaricomycetes; o__Cantharellales; f__Tulasnellaceae; g__unidentified |
| fungi_OTU84 | k__Fungi; p__Basidiomycota; c__Agaricomycetes; o__Cantharellales; f__Tulasnellaceae; g__unidentified |
| fungi_OTU94 | k__Fungi; p__Basidiomycota; c__Agaricomycetes; o__Sebacinales; f__Sebacinaceae; g__unidentified |

**Table S7 Continued**

| OTU | taxonomy |
| --- | --- |
| fungi_OTU63 | k__Fungi; p__Basidiomycota; c__Tremellomycetes; o__unidentified; f__unidentified; g__unidentified |
| fungi_OTU127 | k__Fungi; p__unidentified; c__unidentified; o__unidentified; f__unidentified; g__unidentified |
| fungi_OTU316 | k__Fungi; p__unidentified; c__unidentified; o__unidentified; f__unidentified; g__unidentified |
| fungi_OTU53 | k__Fungi; p__unidentified; c__unidentified; o__unidentified; f__unidentified; g__unidentified |

Abbreviation: Biological soil crusts (BSCs)

**Table S8 The key OTUs/species classification information in** **the co-occurrence network**

| OTU | Taxonomy |
| --- | --- |
| bacteria_OTU33261 | k__Bacteria; p__Acidobacteria; c__[Chloracidobacteria]; o__PK29; f__; g__; s__ |
| bacteria_OTU277 | k__Bacteria; p__Acidobacteria; c__[Chloracidobacteria]; o__PK29; f__; g__; s__ |
| bacteria_OTU10021 | k__Bacteria; p__Acidobacteria; c__[Chloracidobacteria]; o__PK29; f__; g__; s__ |
| bacteria_OTU6251 | k__Bacteria; p__Acidobacteria; c__[Chloracidobacteria]; o__RB41; f__; g__; s__ |
| bacteria_OTU30 | k__Bacteria; p__Acidobacteria; c__[Chloracidobacteria]; o__RB41; f__Ellin6075; g__; s__ |
| bacteria_OTU890 | k__Bacteria; p__Bacteroidetes; c__[Saprospirae]; o__[Saprospirales]; f__Chitinophagaceae; g__; s__ |
| bacteria_OTU71 | k__Bacteria; p__Bacteroidetes; c__Cytophagia; o__Cytophagales; f__Cytophagaceae; g__; s__ |
| bacteria_OTU125 | k__Bacteria; p__Bacteroidetes; c__Cytophagia; o__Cytophagales; f__Cytophagaceae; g__Siphonobacter; s__aquaeclarae |
| bacteria_OTU531 | k__Bacteria; p__Bacteroidetes; c__Flavobacteriia; o__Flavobacteriales; f__Flavobacteriaceae; g__Flavobacterium; s__ |
| bacteria_OTU110 | k__Bacteria; p__Chloroflexi; c__Anaerolineae; o__SBR1031; f__A4b; g__; s__ |
| bacteria_OTU294 | k__Bacteria; p__Chloroflexi; c__Anaerolineae; o__SBR1031; f__A4b; g__; s__ |
| bacteria_OTU108 | k__Bacteria; p__Chloroflexi; c__Anaerolineae; o__SBR1031; f__A4b; g__; s__ |
| bacteria_OTU343 | k__Bacteria; p__Chloroflexi; c__Anaerolineae; o__SBR1031; f__A4b; g__; s__ |
| bacteria_OTU438 | k__Bacteria; p__Chloroflexi; c__Anaerolineae; o__SBR1031; f__A4b; g__; s__ |
| bacteria_OTU1998 | k__Bacteria; p__Chloroflexi; c__Anaerolineae; o__SBR1031; f__oc28; g__; s__ |
| bacteria_OTU773 | k__Bacteria; p__Chloroflexi; c__Anaerolineae; o__SBR1031; f__SJA-101; g__; s__ |
| bacteria_OTU39460 | k__Bacteria; p__Cyanobacteria; c__; o__; f__; g__; s__ |
| bacteria_OTU87 | k__Bacteria; p__Cyanobacteria; c__; o__; f__; g__; s__ |
| bacteria_OTU471 | k__Bacteria; p__Cyanobacteria; c__; o__; f__; g__; s__ |
| bacteria_OTU171 | k__Bacteria; p__Cyanobacteria; c__Nostocophycideae; o__; f__; g__; s__ |
| bacteria_OTU97 | k__Bacteria; p__Cyanobacteria; c__Nostocophycideae; o__Nostocales; f__; g__; s__ |
| bacteria_OTU625 | k__Bacteria; p__Cyanobacteria; c__Nostocophycideae; o__Nostocales; f__Nostocaceae; g__; s__ |
| bacteria_OTU517 | k__Bacteria; p__Cyanobacteria; c__Nostocophycideae; o__Nostocales; f__Scytonemataceae |

**Table S8 Continued**

| OTU | Taxonomy |
| --- | --- |
| bacteria_OTU12298 | k__Bacteria; p__Cyanobacteria; c__Nostocophycideae; o__Nostocales; f__Scytonemataceae; g__Scytonema; s__ |
| bacteria_OTU19176 | k__Bacteria; p__Cyanobacteria; c__Nostocophycideae; o__Nostocales; f__Scytonemataceae; g__Scytonema; s__ |
| bacteria_OTU897 | k__Bacteria; p__Cyanobacteria; c__Nostocophycideae; o__Stigonematales; f__Rivulariaceae; g__Calothrix; s__ |
| bacteria_OTU352 | k__Bacteria; p__Cyanobacteria; c__Oscillatoriophycideae |
| bacteria_OTU11 | k__Bacteria; p__Cyanobacteria; c__Oscillatoriophycideae; o__Oscillatoriales; f__Phormidiaceae |
| bacteria_OTU18766 | k__Bacteria; p__Cyanobacteria; c__Oscillatoriophycideae; o__Oscillatoriales; f__Phormidiaceae; g__Phormidium |
| bacteria_OTU17348 | k__Bacteria; p__Cyanobacteria; c__Oscillatoriophycideae; o__Oscillatoriales; f__Phormidiaceae; g__Phormidium; s__ |
| bacteria_OTU3774 | k__Bacteria; p__Cyanobacteria; c__Oscillatoriophycideae; o__Oscillatoriales; f__Phormidiaceae; g__Phormidium; s__ |
| bacteria_OTU84 | k__Bacteria; p__Cyanobacteria; c__Oscillatoriophycideae; o__Oscillatoriales; f__Phormidiaceae; g__Phormidium; s__ |
| bacteria_OTU43 | k__Bacteria; p__Cyanobacteria; c__Oscillatoriophycideae; o__Oscillatoriales; f__Phormidiaceae; g__Phormidium; s__ |
| bacteria_OTU7224 | k__Bacteria; p__Cyanobacteria; c__Synechococcophycideae |
| bacteria_OTU16659 | k__Bacteria; p__Cyanobacteria; c__Synechococcophycideae; o__Pseudanabaenales; f__Pseudanabaenaceae |
| bacteria_OTU25887 | k__Bacteria; p__Cyanobacteria; c__Synechococcophycideae; o__Pseudanabaenales; f__Pseudanabaenaceae; g__Leptolyngbya; s__ |
| bacteria_OTU393 | k__Bacteria; p__Cyanobacteria; c__Synechococcophycideae; o__Pseudanabaenales; f__Pseudanabaenaceae; g__Leptolyngbya; s__ |
| bacteria_OTU4010 | k__Bacteria; p__Cyanobacteria; c__Synechococcophycideae; o__Pseudanabaenales; f__Pseudanabaenaceae; g__Leptolyngbya; s__ |
| bacteria_OTU8422 | k__Bacteria; p__Cyanobacteria; c__Synechococcophycideae; o__Pseudanabaenales; f__Pseudanabaenaceae; g__Leptolyngbya; s__ |
| bacteria_OTU3877 | k__Bacteria; p__Cyanobacteria; c__Synechococcophycideae; o__Pseudanabaenales; f__Pseudanabaenaceae; g__Leptolyngbya; s__ |
| bacteria_OTU54 | k__Bacteria; p__Cyanobacteria; c__Synechococcophycideae; o__Pseudanabaenales; f__Pseudanabaenaceae; g__Leptolyngbya; s__ |

**Table S8 Continued**

| OTU | Taxonomy |
| --- | --- |
| bacteria_OTU639 | k__Bacteria; p__Cyanobacteria; c__Synechococcophycideae; o__Pseudanabaenales; f__Pseudanabaenaceae; g__Leptolyngbya; s__ |
| bacteria_OTU1257 | k__Bacteria; p__Cyanobacteria; c__Synechococcophycideae; o__Pseudanabaenales; f__Pseudanabaenaceae; g__Leptolyngbya; s__ |
| bacteria_OTU1355 | k__Bacteria; p__Cyanobacteria; c__Synechococcophycideae; o__Pseudanabaenales; f__Pseudanabaenaceae; g__Leptolyngbya; s__ |
| bacteria_OTU476 | k__Bacteria; p__Cyanobacteria; c__Synechococcophycideae; o__Pseudanabaenales; f__Pseudanabaenaceae; g__Leptolyngbya; s__ |
| bacteria_OTU748 | k__Bacteria; p__Gemmatimonadetes; c__Gemm-3; o__; f__; g__; s__ |
| bacteria_OTU521 | k__Bacteria; p__Gemmatimonadetes; c__Gemmatimonadetes; o__Gemmatimonadales; f__; g__; s__ |
| bacteria_OTU1342 | k__Bacteria; p__Planctomycetes; c__Phycisphaerae |
| bacteria_OTU1007 | k__Bacteria; p__Planctomycetes; c__Phycisphaerae; o__WD2101; f__; g__; s__ |
| bacteria_OTU1048 | k__Bacteria; p__Planctomycetes; c__Planctomycetia; o__Gemmatales; f__Gemmataceae; g__Gemmata; s__ |
| bacteria_OTU1174 | k__Bacteria; p__Planctomycetes; c__Planctomycetia; o__Gemmatales; f__Gemmataceae; g__Gemmata; s__ |
| bacteria_OTU768 | k__Bacteria; p__Planctomycetes; c__Planctomycetia; o__Gemmatales; f__Gemmataceae; g__Gemmata; s__ |
| bacteria_OTU380 | k__Bacteria; p__Proteobacteria; c__Alphaproteobacteria; o__Caulobacterales; f__Caulobacteraceae; g__; s__ |
| bacteria_OTU142 | k__Bacteria; p__Proteobacteria; c__Alphaproteobacteria; o__Rhodobacterales; f__Rhodobacteraceae; g__Rubellimicrobium; s__ |
| bacteria_OTU562 | k__Bacteria; p__Proteobacteria; c__Alphaproteobacteria; o__Rhodospirillales; f__Acetobacteraceae |
| bacteria_OTU1263 | k__Bacteria; p__Proteobacteria; c__Alphaproteobacteria; o__Rhodospirillales; f__Acetobacteraceae; g__; s__ |
| bacteria_OTU1238 | k__Bacteria; p__Proteobacteria; c__Alphaproteobacteria; o__Rickettsiales; f__; g__; s__ |
| bacteria_OTU17 | k__Bacteria; p__Proteobacteria; c__Alphaproteobacteria; o__Sphingomonadales; f__Erythrobacteraceae; g__; s__ |
| bacteria_OTU14513 | k__Bacteria; p__Proteobacteria; c__Alphaproteobacteria; o__Sphingomonadales; f__Sphingomonadaceae |
| bacteria_OTU8543 | k__Bacteria; p__Proteobacteria; c__Alphaproteobacteria; o__Sphingomonadales; f__Sphingomonadaceae; g__; s__ |

**Table S8 Continued**

| OTU | Taxonomy |
| --- | --- |
| bacteria_OTU134 | k__Bacteria; p__Proteobacteria; c__Alphaproteobacteria; o__Sphingomonadales; f__Sphingomonadaceae; g__Sphingomonas |
| bacteria_OTU31164 | k__Bacteria; p__Proteobacteria; c__Alphaproteobacteria; o__Sphingomonadales; f__Sphingomonadaceae; g__Sphingomonas; s__changbaiensis |
| bacteria_OTU1252 | k__Bacteria; p__Proteobacteria; c__Deltaproteobacteria; o__Myxococcales; f__Cystobacterineae; g__; s__ |
| bacteria_OTU1094 | k__Bacteria; p__Verrucomicrobia; c__[Spartobacteria]; o__[Chthoniobacterales]; f__[Chthoniobacteraceae]; g__; s__ |
| bacteria_OTU1163 | k__Bacteria; p__Verrucomicrobia; c__[Spartobacteria]; o__[Chthoniobacterales]; f__[Chthoniobacteraceae]; g__; s__ |
| bacteria_OTU234 | k__Bacteria; p__Verrucomicrobia; c__Opitutae; o__Opitutales; f__Opitutaceae; g__Opitutus; s__ |
| fungi_OTU25 | k__Fungi; p__Ascomycota; c__Dothideomycetes; o__Pleosporales; f__unidentified; g__unidentified |
| fungi_OTU145 | k__Fungi; p__Ascomycota; c__Dothideomycetes; o__Pleosporales; f__unidentified; g__unidentified |
| fungi_OTU34 | k__Fungi; p__Ascomycota; c__Lecanoromycetes; o__Lecanorales; f__Lecanorales_fam_Incertae_sedis; g__Leprocaulon |
| fungi_OTU69 | k__Fungi; p__Ascomycota; c__Pezizomycotina_cls_Incertae_sedis; o__Pezizomycotina_ord_Incertae_sedis; f__Pezizomycotina_fam_Incertae_sedis; g__Knufia |
| fungi_OTU86 | k__Fungi; p__Ascomycota; c__Pezizomycotina_cls_Incertae_sedis; o__Pezizomycotina_ord_Incertae_sedis; f__Pezizomycotina_fam_Incertae_sedis; g__Ochroconis |
| fungi_OTU380 | k__Fungi; p__Ascomycota; c__Sordariomycetes; o__Sordariomycetidae_ord_Incertae_sedis; f__Glomerellaceae; g__Colletotrichum |
| fungi_OTU84 | k__Fungi; p__Basidiomycota; c__Agaricomycetes; o__Cantharellales; f__Tulasnellaceae; g__unidentified |
| fungi_OTU63 | k__Fungi; p__Basidiomycota; c__Tremellomycetes; o__unidentified; f__unidentified; g__unidentified |
